# Preclinical drug screen identifies WEE1 inhibitor and vinca alkaloid as a combination treatment concept for Li-Fraumeni syndrome medulloblastoma

**DOI:** 10.1016/j.isci.2025.114564

**Published:** 2025-12-29

**Authors:** Anna S. Kolodziejczak, Florian Selt, Heike Peterziel, Nora Jamaladdin, Norman Mack, Kendra Maaß, Chris Meulenbroeks, Romain Sigaud, Christel Herold-Mende, Ahmed El Damaty, Jürgen Burhenne, Shunya Ohmura, Tim Holland-Letz, Lena M. Kutscher, Aurélie Ernst, Pei-Chi Wei, Thomas G.P. Grünewald, Ina Oehme, Marcel Kool, David T.W. Jones, Kristian W. Pajtler, Christian P. Kratz, Stefan M. Pfister, Olaf Witt, Till Milde

**Affiliations:** 1 Hopp Children’s Cancer Center (KiTZ), 69120 Heidelberg, Germany; 2 CCU Pediatric Oncology, German Cancer Research Center (DKFZ) and German Cancer Consortium (DKTK), 69120 Heidelberg, Germany; 3 Department of Pediatric Oncology, Hematology, Immunology and Pulmonology, Heidelberg University Hospital, 69120 Heidelberg, Germany; 4 Heidelberg University, 69117 Heidelberg, Germany; 5 Pediatric Neurooncology, German Cancer Research Center (DKFZ) and German Cancer Consortium (DKTK), 69120 Heidelberg, Germany; 6 Princess Máxima Center for Pediatric Oncology, 3584 CS Utrecht, the Netherlands; 7 University Hospital Jena, Department of Pediatrics and Adolescent Medicine, Friedrich Schiller University Jena, 07747 Jena, Germany; 8 Comprehensive Cancer Center Central Germany (CCCG), 07743 Jena, Germany; 9 Division of Experimental Neurosurgery, Department of Neurosurgery, University of Heidelberg, Medical Faculty Heidelberg, 69120 Heidelberg, Germany; 10 Pediatric Neurosurgery Division, Department of Neurosurgery, Heidelberg University Hospital, 69120 Heidelberg, Germany; 11 Department of Clinical Pharmacology and Pharmacoepidemiology, Medical Faculty Heidelberg, Heidelberg University Hospital, 69120 Heidelberg, Germany; 12 Division of Translational Pediatric Sarcoma Research, German Cancer Research Center (DKFZ), German Cancer Consortium (DKTK), 69120 Heidelberg, Germany; 13 National Center for Tumor Diseases (NCT), 69120 Heidelberg, Germany; 14 Department of Biostatistics, German Cancer Research Center (DKFZ), 69120 Heidelberg, Germany; 15 Developmental Origins of Pediatric Cancer Junior Research Group, German Cancer Research Center (DKFZ), 69120 Heidelberg, Germany; 16 Group Genome Instability in Tumors, German Cancer Research Center (DKFZ), 69120 Heidelberg, Germany; 17 German Cancer Consortium (DKTK), DKFZ, Core Center, 69120 Heidelberg, Germany; 18 Brain Mosaicism and Tumorigenesis, German Cancer Research Center, 69120 Heidelberg, Germany; 19 Institute of Pathology, Heidelberg University Hospital, 69120 Heidelberg, Germany; 20 National Center for Tumor Diseases (NCT), 69120 Heidelberg, Germany; 21 University Medical Center Utrecht, 3584 CX Utrecht, the Netherlands; 22 Pediatric Glioma Research, German Cancer Research Center (DKFZ) and German Cancer Consortium (DKTK), 69120 Heidelberg, Germany; 23 Paediatric Haematology and Oncology, Hannover Medical School, 30625 Hannover, Germany; 24 Division of Pediatric Neurooncology, German Cancer Research Center (DKFZ) and German Cancer Consortium (DKTK), 69120 Heidelberg, Germany

**Keywords:** Biological sciences, Cancer systems biology, Natural sciences, Pharmacology, Systems biology

## Abstract

Li-Fraumeni syndrome (LFS) is characterized by constitutional pathogenic *TP53* mutation and increased risk of cancer development, including Sonic Hedgehog-activated medulloblastoma (SHH-MB). In LFS patients, radiation and DNA-damaging agents can exhibit lower efficiency and cause secondary malignancies. To identify efficacious, safe chemotherapeutic approaches for LFS-associated SHH-MB, 333 compounds were screened in *in vitro* TP53^mut^ brain tumor cell lines. The combination of WEE1 inhibitor adavosertib and vinca alkaloid vincristine demonstrated the highest activity, which was validated in TP53^mut^ SHH-MB patient-derived organoids. Low genotoxicity of these compounds was determined *in vitro* in LFS fibroblasts, and *in vivo* in the LFS mouse model. Despite the drugs’ limited efficacy in the *in vivo* PDX model, WEE1 knockdown led to significant growth reduction in *in vitro* and *in vivo* TP53^mut^ SHH-MB models. Our findings identify WEE1 as a promising target in LFS SHH-MB, suggesting its inhibition combined with vincristine treatment as a potential chemotherapeutic strategy.

## Highlights

• Drug screen identified adavosertib and vincristine as top hits against LFS SHH-MB
• Patient expression signatures supported TP53^mut^ SHH-MB sensitivity to adavosertib
• Adavosertib and vincristine combination showed low toxicity in LFS mouse model
• Knockdown of adavosertib target WEE1 reduced growth of TP53^mut^ SHH-MB models

## Graphical abstract

**Figure:**
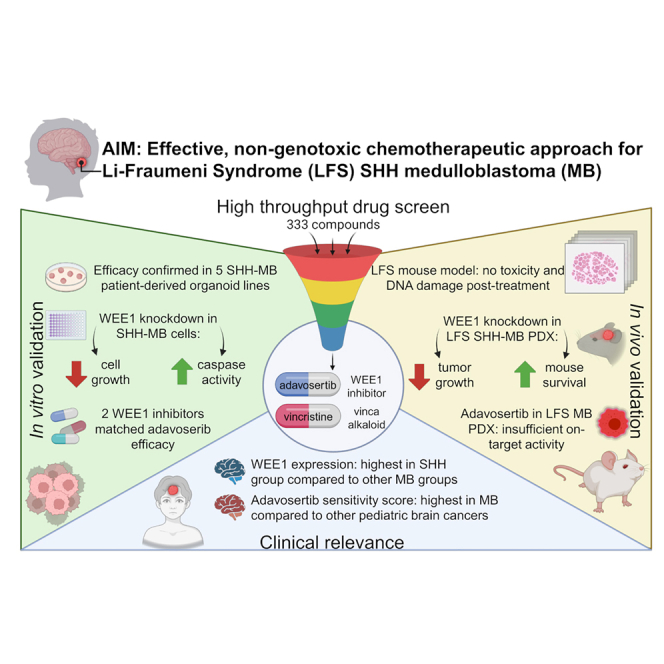

## Introduction

Li-Fraumeni syndrome (LFS), first described by Frederick Pei Li and Joseph F. Fraumeni, Jr. in 1969,^1^ is a frequently diagnosed cancer predisposition syndrome (CPS) with incidence rate between 1:5,000 and 1:20,000 worldwide.^2^^,^^3^ In the majority of affected individuals, constitutional mutations of the *TP53* gene, encoding an important tumor suppressor, are found.^4^^,^^5^ The cancer spectrum in LFS patients mostly consists of sarcoma, breast cancer, adrenocortical carcinoma, and brain tumors.^6^^,^^7^^,^^8^ In pediatrics, *TP53* mutated (TP53^mut^) Sonic Hedgehog-activated medulloblastoma (SHH-MB), is the most common LFS-associated brain tumor that renders a dismal prognosis.^9^^,^^10^

Challenges in cancer therapy of LFS patients extend beyond an increased cancer risk. While several detailed screening and surveillance protocols for individuals with LFS have been tested and implemented into clinical practice,^11^^,^^12^^,^^13^ a uniform agreement on the therapeutic anti-neoplastic strategy for LFS-associated cancer is lacking. The majority of conventional cancer treatment regimens rely on chemotherapy and radiation causing DNA damage. Extensive and persistent DNA strand breaks frequently trigger cell death via apoptosis. Hence, due to the underlying defect in response mechanism to DNA damage, LFS patients commonly show poor response to this conventional treatment.^6^^,^^11^^,^^14^ Moreover, multiple reports support the causal relationship between DNA damaging agents and secondary malignancies in LFS patients.^15^^,^^16^^,^^17^ Thus, novel treatments for LFS-tumors that are both effective and non-toxic to non-cancerous tissues are urgently needed. Combination treatments are considered more beneficial than single drugs, as application of two or more drugs enhances efficacy by targeting multiple pathways, decreases risk of cross resistance and addresses intra- and intertumoral heterogeneity.^18^

In this study, we searched for drugs effective in LFS-associated MB tumors that efficiently reduce viability of cancer cells without causing genotoxic stress in non-cancerous tissues. First, a comprehensive drug library was screened *in vitro* in two TP53^mut^ brain cancer cell lines and non-cancerous LFS fibroblasts. Next, the results were validated *in vivo* in TP53^mut^ SHH-MB PDX and the LFS mouse model. The identified novel chemotherapeutic approach may broaden therapeutic options for LFS-associated SHH-MB and improve patient outcomes in future clinical trials.

## Results

### *In vitro* drug screen identifies several effective drug groups in LFS-associated brain cancer cell lines

**Figure 1:**
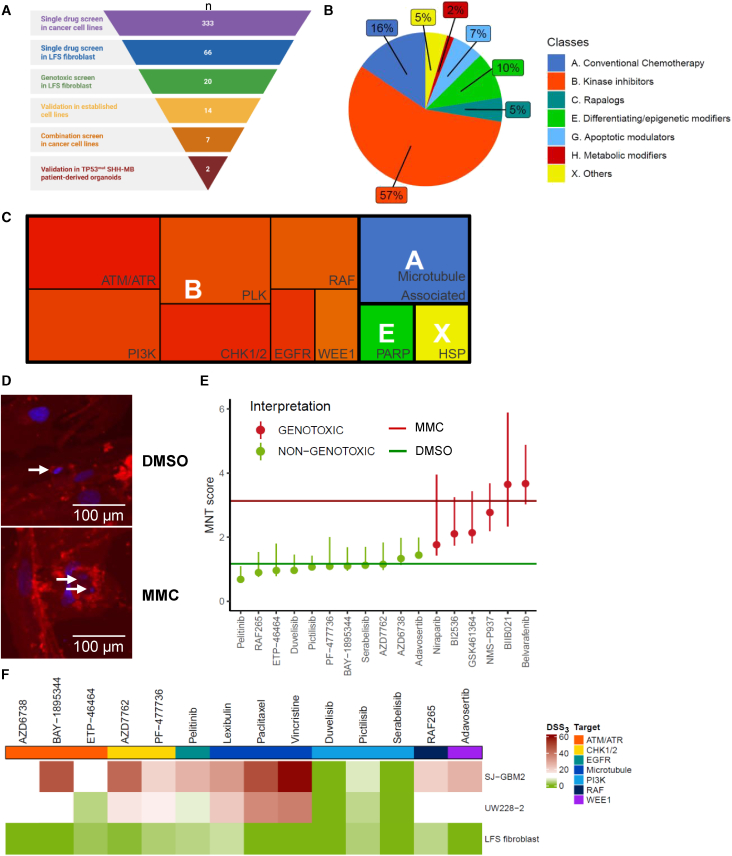
Single drug screen in LFS *in vitro* cell line models (A) Drug screening pipeline (*n*, number of compounds; TP53mut SHH-MB, TP53-mutated SHH-activated MB) (Created by BioRender.com). (B) Drug library composition (see also Table S1). (C) Targets of single drug screen hits (drug classes: A, conventional chemotherapy; B, kinase inhibitors; E, differentiating/epigenetic modulator; X, other): the size of each rectangle reflects the number of compounds in a given group of drugs targeting a specific pathway, function or protein, names in the lower right corners correspond to drug targets (see also Figure S1A). (D) Micronucleus assay (MNT) images of LFS fibroblasts exposed to DMSO (negative control) and mitomycin C (MMC, positive control); arrows indicate micronuclei; scale bars, 100 μm (E) MNT scores of single drug screen hits: horizontal lines: MMC, median MNT score for the positive control (MMC); DMSO, median MNT score for the negative control (DMSO); vertical lines: median MNT scores with 95% confidence interval for tested compounds (see also Figure S1B and Table S3). (F) Results of “validation in established cell lines” step performed in *in vitro* LFS models (DSS_3_, Drug Sensitivity Score 3): data are represented as mean values (see also Table 1 and Figure S1C).

A high throughput drug screen was performed to identify an effective and non-toxic drug combination (Figure 1A). The library for drug screening purpose consisted of 333 compounds targeting 61 different human proteins (Figure 1B). More than half of compounds (56.8%) belonged to the class of kinase inhibitors. Furthermore, the library included conventional chemotherapeutics (15.6%), rapalogs (5.1%), differentiating or epigenetic modifiers (9.9%), apoptotic modulators (6.6%) and metabolic modifiers (1.5%), and others (4.5%) (Table S1). The majority of compounds (79%) was either approved or tested in clinical trials in year 2025 (Table S1).

A single-replicate limited concentration range single drug screen was performed in two TP53^mut^ brain tumor cell lines, SJGBM-2 (*TP53* p.R273C) and UW228-2 (*TP53* p.T155N) (Figure 1A and Table S2). Drug Sensitivity Score 3 (DSS_3_)^19^ was chosen as a parameter for determining the effectiveness. If DSS_3_ was equal or above 20 in both cell lines, a compound was considered effective and a positive hit. This criterium was fulfilled by 66 compounds (Figures 1A, 1C; Table S1).

Comparing results in the two brain cancer lines, UW228-2 was significantly less sensitive overall than SJ-GBM2 (median DSS_3_ 9.5 and 1.3, respectively; Wilcoxon test: *p* < 0.05). Differences in DSS_3_ values were statistically significant for drug classes A (conventional chemo), B (kinase inhibitors), E (differentiating or epigenetic modifiers), and G (apoptotic modulators). Regarding specific drug targets, the greatest differences in median DSS_3_ values were observed for inhibitors targeting PI3K, AKT, and DNA-PK—where UW228-2 cells exhibited higher sensitivity compared to SJ-GBM2—and for inhibitors targeting E3 ligase, Aurora kinase, and CDKs, for which SJ-GBM2 cells demonstrated greater sensitivity than UW228-2.

### Kinase inhibitors and microtubule-associated compounds have minor cytotoxic and genotoxic effects in LFS non-cancerous cells *in vitro*

The 66 most effective compounds were tested in non-cancerous LFS fibroblasts under the same conditions as the cancer cell lines. Twenty-four compounds, for which DSS_3_ did not exceed 10 and dDSS_3_ (difference between DSS_3_ in cancerous and non-cancerous cell lines) was greater than 20, were determined as non-cytotoxic for non-cancerous cells (Figures 1C and S1A; Table S1). Four compounds (clofarabine, floxuridine, azathioprine, and methotrexate) were excluded from the further testing due to their previously described DNA damaging properties in LFS *in vitro* and *in vivo* models,^20^^,^^21^ resulting in 20 compounds screened for genotoxicity.

To test drug hits from the cytotoxicity screen for potential genotoxic effects, the micronucleus test (MNT) was performed as a genotoxic screen to measure micronuclei formation upon treatment in LFS fibroblasts (Figure 1D). All compounds for which MNT scores were significantly different from the positive control mitomycin C (MMC) (*p* < 0.05) were defined non-genotoxic. Out of 20 compounds, 11 showed no genotoxic effects in LFS fibroblasts (Figure 1E and Table S3). For microtubule-associated compounds, micronucleation caused by chromosomal breakage could not be distinguished from that resulting from their known mechanism of action - interaction with tubulin leading to mitotic spindle dysfunction (Figure S1B and Table S3). These three microtubule-associated compounds were nonetheless included in further tests, based on previous studies demonstrating that these compounds do not induce significant DNA damage leading to tumorigenesis in TP53^mut^ cells.^20^^,^^21^^,^^22^

The remaining 14 drug hits were re-evaluated (Figure 1F) in the two TP53^mut^ brain tumor cell lines (SJGBM-2 and UW228-2) and LFS fibroblasts in triplicates using a wider concentration range (0.1–10,000 nM). Compounds that did not reach the maximum effect (*E*_max_) > 50% in cancer cell lines were subsequently excluded. Eight drugs reached an *E*_max_ > 50%: two ATM/ATR inhibitors (AZD6738 and BAY-1895344), two CHK1/2 inhibitors (PF-477736 and AZD7762), WEE1 inhibitor (adavosertib), and three microtubule-associated compounds (lexibulin, paclitaxel, and vincristine) (Figures 1F and S1C and Table 1).

**Table 1:** Validated single drug screen hits in established *in vitro* LFS cancerous and non-cancerous cell lines

Compound	Target	SJ-GBM2 DSS_3_	SJ-GBM2 *E*_max_	UW228-2 DSS_3_	UW228-2 *E*_max_	LFS fibroblast DSS_3_
AZD6738	ATM/ATR	12.7	94.8	10.5	67.5	0.0
BAY-1895344	ATM/ATR	46.2	95.3	12.8	74.1	0.0
AZD7762	CHK1/2	42.1	99.5	15.5	97.0	0.9
PF-477736	CHK1/2	19.3	99.2	13.5	77.8	3.5
Lexibulin	microtubules	31.5	92.4	21.4	64.8	5.6
Paclitaxel	microtubules	47.8	90.6	35.1	87.5	0.0
Vincristine	microtubules	59.5	92.5	37.4	52.2	0.0
Adavosertib	WEE1	26.1	98.1	10.4	89.1	0.0

DSS_3_, Drug Sensitivity Score 3; *E*_max_, maximum effect.

### The combination of WEE1 inhibitor adavosertib and vincristine decreases cell viability in LFS-associated brain tumor cells *in vitro*

**Figure 2:**
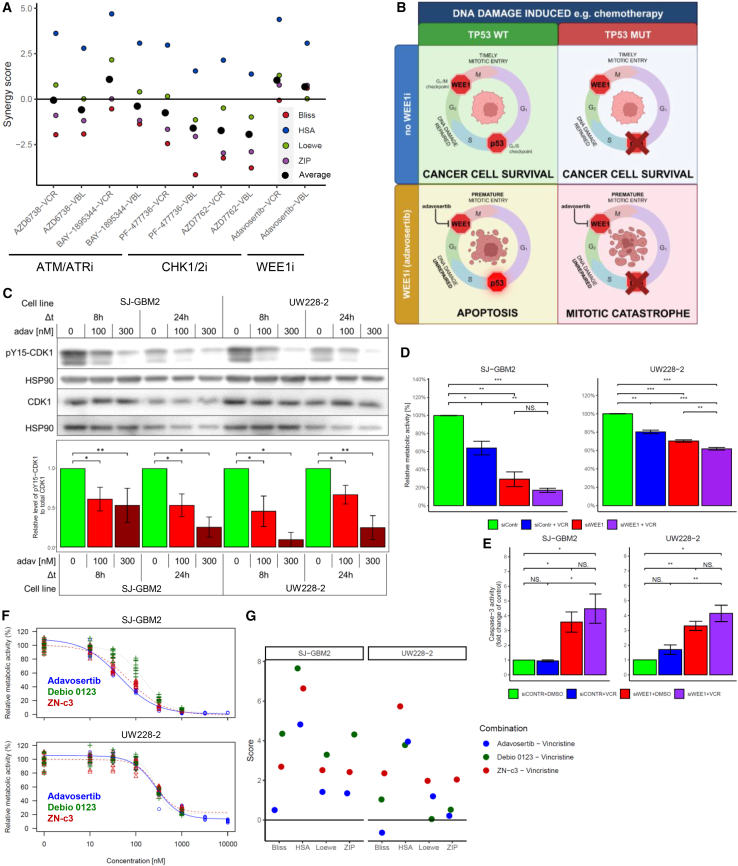
Drug combination screening and drug hits validation in *in vitro* TP53^mut^ brain cancer cell models (A) Average synergy scores estimated for combinations of cell cycle checkpoint kinase inhibitors and micronucleus-associated compounds across TP53^mut^ brain cancer cell lines; black points indicate the average value across all calculated synergy scores (ATM/ATRi, ATM/ATR inhibitors; CHK1/2i, CHEK1/CHEK2 inhibitors; WEE1i, WEE1 inhibitor; VBL, vinblastine; VCR, vincristine) (see also Table S4). (B) Model representing mechanism of action of adavosertib in TP53^wt^ and TP53^mut^ cancer cells exposed to DNA damaging agents (Created with BioRender.com). (C) Adavosertib (adav) decreases CDK1 phosphorylation levels in SJ-GBM2 and UW228-2 cell lines; Student’s *t* test was used for statistical analysis, data are represented as mean ± SEM (Δ*t*, exposure time; ∗*p* < 0.05; ∗∗*p* < 0.01). (D) WEE1 knockdown (KD) and vincristine (VCR) effect on metabolic activity of *in vitro* TP53^mut^ brain cancer cell lines: data are represented as mean ± SEM (siContr, control siRNA; siWEE1, WEE1 siRNA; NS, not significant; ∗*p* < 0.05; ∗∗*p* < 0.01) (see also Figure S2C). (E) WEE1 KD and VCR effect on *in vitro* caspase-3 activity in TP53^mut^ brain cancer cell lines: data are represented as mean ± SEM (NS, not significant; ∗*p* < 0.05; ∗∗*p* < 0.01) (see also Figure S2C). (F) Dose-response curves of WEE1 inhibitors (adavosertib, Debio 0123, and ZN-c3) in *in vitro* TP53^mut^ brain cancer cell lines: data are represented as mean ± SEM. (G) Synergy scores of vincristine and WEE1 inhibitors combination treatment in *in vitro* TP53^mut^ brain cancer cell lines: data are represented as mean.

Combinations of the selected compounds (five kinase inhibitors and two microtubule-associated compounds) were investigated in the previously used cancer cell lines (SJ-GBM2 and UW228-2) (Figure 2A). Due to their low clinical relevance for pediatric brain tumor treatment, the microtubule-associated drugs lexibulin and paclitaxel were replaced in the combination screen by vinblastine, a vinca alkaloid commonly used in MB therapy. ATM, ATR, CHK1, CHK2, and WEE1 inhibitors were not combined with each other due to targeting of the same cell cycle checkpoint pathway. Combinations with both vinblastine and vincristine were additive and never antagonistic by synergy score (Figure 2A and Table S4). Based on the consensus ranking the most efficient combinations were adavosertib plus vincristine and BAY-1895344 plus vincristine (Table S4). Both drugs had similar effects in LFS SHH-MB model (UW228-2) and non-malignant control (LFS fibroblasts): DSS in UW228-2 for adavosertib and BAY-1895344 were 10.4 and 12.8, respectively, whereas in LFS fibroblasts DSS was 0 for both compounds (Figure S1D).

To increase clinical relevance we analyzed published results of clinical trials^23^^,^^24^^,^^25^ and reported blood tumor barrier (BTB) penetration^26^^,^^27^ of the WEE1 inhibitor adavosertib and the ATM/ATR inhibitor BAY-1895344. Considering the total number of clinical trials (adavosertib: 61, BAY-1895344: 9, access date: 26.05.2023), the number of clinical trials including pediatric patients (adavosertib: 5, BAY-1895344: 1, access date: 26.05.2023) as well as reported intra-tumoral drug concentrations (adavosertib in GBM patients: 858 nM,^26^ BAY-1895344 in mice: 495 nM^27^) we concluded that adavosertib had a higher potential than BAY-1895344 for immediate implementation as part of LFS SHH-MB treatment regimen. Consequently, adavosertib was chosen as a combination partner for all further experiments.

Adavosertib directly targets WEE1, a kinase responsible for G2/M cell cycle checkpoint transition. Inhibition of WEE1 in TP53-negative cancer cells leads to persisting DNA damage, replication stress and in consequence mitotic catastrophe^28^^,^^29^^,^^30^ (Figure 2B). We demonstrated on-target activity of adavosertib in two cell lines, showing reduced levels of CDK1 phosphorylation following adavosertib exposure (Figure 2C).

### TP53^mut^ SHH-MB growth is dependent on WEE1 activity

To confirm target dependence on WEE1 activity of TP53^mut^ brain tumor cell growth, endogenous WEE1 protein was knocked down (KD) in two TP53^mut^ brain tumor cell lines (SJGBM-2 and UW228-2). Upon siRNA-mediated knockdown (Figure S2A) cells transfected with siRNA targeting WEE1 showed significantly reduced growth compared to the control (SJ-GBM2: mean viability: 25.5%, *p* < 0.05, UW228-2: mean viability: 70.3%, *p* < 0.01) (Figure 2D). The combination of low concentration of vincristine (VCR, 0.5 nM) and WEE1 KD in UW228-2 was significantly more effective than single treatments (VCR: mean viability: 80.2%, siWEE1+VCR: mean viability: 61.8%, VCR vs. siWEE1+VCR: *p* < 0.001, siWEE1 vs. siWEE1+VCR: *p* < 0.01) (Figure 2D). In SJ-GBM2 cell line the difference in viability was significant only when comparing single treatment with vincristine and combination treatment (VCR: mean viability: 62.9%, siWEE1+VCR: mean viability: 16.2%, VCR vs. siWEE1+VCR: *p* < 0.01, siWEE1 vs. siWEE1+VCR: *p* > 0.05) (Figure 2D). Bliss synergy score was −0.2 in SJ-GBM2 and -5.14 in UW228-2, suggesting an additive effect in both cell lines.

Knockdown of WEE1 kinase also significantly activated caspase-3 in both SJ-GBM2 and UW228-2 cell lines. Treatment with VCR did not influence caspase-3 activity, in line with the compound’s cytostatic rather than cytotoxic effect in TP53^mut^ cancer cells, and did not reduce the pro-apoptotic effect of WEE1 KD (Figure 2E).

Two additional WEE1 inhibitors, Debio 0123 and ZN-c3, were tested in TP53^mut^ brain cancer cell lines. The efficacy of these compounds was comparable with efficacy of adavosertib (IC_50_: SJ-GBM2: adavosertib: 43.4 nM, Debio 0123: 147.1 nM, ZN-c3: 69.7 nM, UW228-2: adavosertib: 272.7 nM, Debio 0123: 270.5 nM, ZN-c3: 271.1 nM) (Figure 2F). Similarly to adavosertib, Debio 0123 and ZN-c3 showed additive interaction in combination with vincristine (Figure 2G).

### Validation of drug hits was performed in *in vitro* TP53^mut^ SHH-MB patient-derived organoids

**Figure 3:**
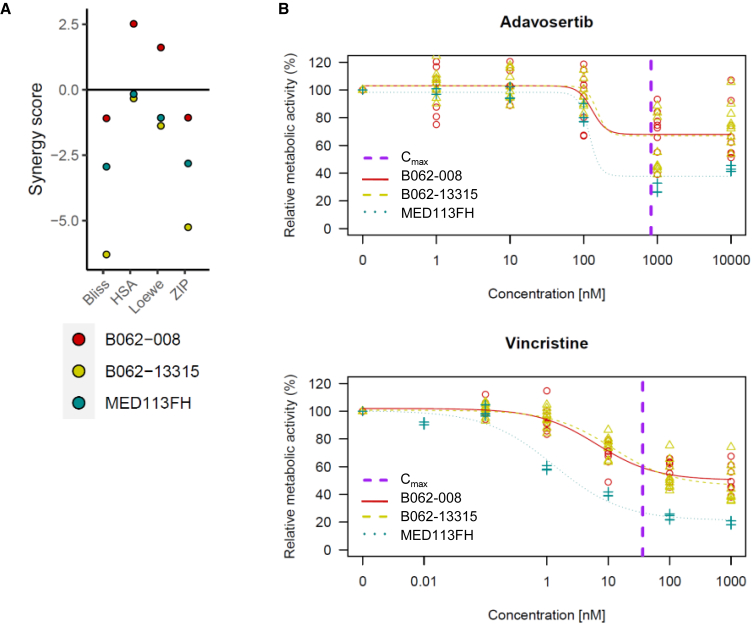
Drug hits validation in *in vitro* TP53^mut^ SHH-MB PDO cultures (A) Synergy scores estimated for adavosertib and vincristine combination in *in vitro* TP53^mut^ SHH-MB PDO cultures (see also Figure S2A and Table S5). (B) Dose-response curves of adavosertib and vincristine in *in vitro* TP53^mut^ SHH-MB PDO cultures: data are represented as mean ± SEM (literature sources of *C*_max_ values are indicated in Table S1).

Subsequently, the combination of adavosertib and vincristine was validated in TP53^mut^ SHH-MB patient-derived organoids (PDOs), to increase the clinical relevance of our findings. In all three PDO models tested, additive and no antagonistic effects were observed, with synergy scores ranging from −6.35 to 2.52 (Figures 3A and S2B), confirming our findings from established cell lines. Of note, the efficacy of vincristine as a single compound with respect to reduction of metabolic activity in PDOs was smaller (mean IC_50_: 6.8 nM, range: 1.38–11.9 nM) (Figure 3B and Table S5) compared to the established cell lines (SJ-GBM2 IC_50_: 1.1 nM, UW228-2 IC_50_: 0.9 nM). In case of adavosertib, the PDOs exhibited higher IC_50_ values (mean IC_50_: 166.5 nM, range: 117.4–215.3 nM) than SJ-GBM2 (IC_50_: 43.4 nM), and lower than UW228-2 (IC_50_: 272.7 nM). Such differences are indeed often seen in drugs highly dependent on cell cycling, relevant for both compounds, when comparing established 2D cell lines (in this case SJ-GBM2 with a 3- to 8-fold lower IC_50_) with 3D PDOs: organoid models tend to proliferate and cycle at a slower rate, show different expression patterns and impaired drug penetration into the 3D structure^31^^,^^32^ resulting in lower sensitivity of PDOs *ex vivo*. In summary, an additive effect of adavosertib and vincristine treatment on metabolic activity was observed in all three TP53^mut^ SHH-MB PDOs tested.

### WEE1 expression and adavosertib sensitivity score are high in SHH-MB tumors compared to other pediatric brain tumor entities

**Figure 4:**
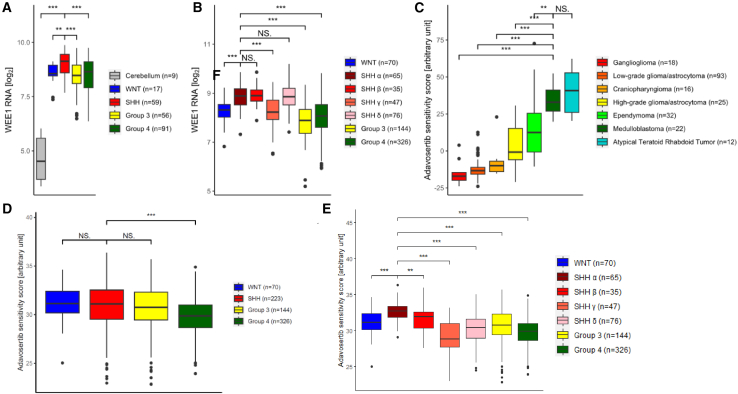
WEE1 as a therapeutic target in pediatric brain tumor entities (A) WEE1 mRNA expression in MB tumors by methylation group (MB dataset: Northcott [*n* = 491],^34^ cerebellum dataset: Roth [*n* = 9]^33^). (B) WEE1 mRNA expression in MB subgroups (Cavalli [*n* = 763]^35^). (C) Adavosertib sensitivity score in pediatric brain tumors (Petralia [*n* = 218]^36^). (D) Adavosertib sensitivity score in MB groups (Cavalli [*n* = 763]^35^). (E) Adavosertib sensitivity score in MB tumors by methylation group (Cavalli [*n* = 763]^35^) (see also Figure S2B). Data are represented as mean ± SEM (Student’s *t* test significance levels: NS, not significant; ∗*p* < 0.05; ∗∗*p* < 0.01; ∗∗∗*p* < 0.001).

Target and predictive biomarker expression was studied in published datasets of primary pediatric brain tumors. Analysis of Roth^33^ and Northcott^34^ cohort mRNA expression data showed that WEE1 mRNA levels are significantly higher in MB tumors compared to normal cerebellum (Figure 4A), and highest in SHH-MB among all MB (*p* < 0.01) (Figure 4A). Within SHH-MB, SHH-α (SHH_3, which contains the LFS-MB cases), -β (SHH_1), and –δ (SHH_4) had the highest expression of WEE1 mRNA (Figure 4B).

Since previous studies have shown an ambiguous relation between WEE1 expression and adavosertib sensitivity in different cancer types,^37^^,^^38^^,^^39^ we strengthened our analysis using a validated signature reflecting adavosertib sensitivity for pediatric tumors.^39^ Primary MB had significantly higher adavosertib sensitivity score (AdSS) than most pediatric brain tumors (*p* < 0.01), only similar to atypical teratoid rhabdoid tumors (ATRT) (n.s.) (Figure 4C). While the AdSS for SHH-MB was similar to WNT and Group 3 and higher compared to Group 4 (*p* < 0.001) (Figure 4D), the SHHα (SHH_3) subgroup (which includes the LFS-MB cases) had the highest AdSS among all SHH-MB subgroups (*p* < 0.01) (Figure 4E). *TP53* mutation alone was not associated with higher sensitivity score in MB (Figure S2C), confirming data from clinical trials including adavosertib, which showed that TP53^mut^ was not a predictive biomarker.^40^

### Reduction of WEE1 activity limits growth of TP53^mut^ SHH-MB PDX models *in vivo*

**Figure 5:**
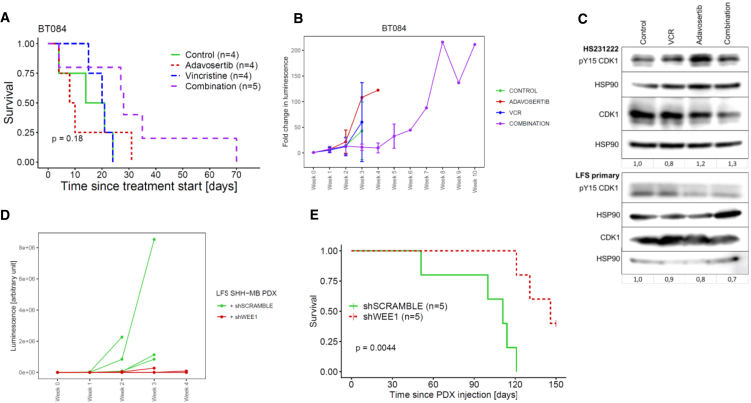
*In vivo* validation of adavosertib and vincristine (VCR) in LFS SHH-MB models (A) Survival of mice injected with BT084 patient-derived xenograft (PDX) model during treatment with adavosertib and VCR; log rank test was used for statistical analysis. (B) Tumor growth dynamics of BT084 PDX model during treatment with adavosertib and vincristine (VCR): data are represented as mean ± SEM. (C) Phospho (p)-CDK1 in LFS SHH-MB PDX cells (HS231222 and LFS primary) following *in vivo* treatment with adavosertib and VCR: numbers below the blot represent normalized fold change relative to non-treated control. (D) Tumor growth dynamics of LFS MB PDX models expressing WEE1 shRNA (shWEE1) and control shRNA (shSCRAMBLE). (E) Survival of mice injected with LFS MB PDX models expressing shWEE1 and shSCRAMBLE; log rank test was used for statistical analysis.

*In vivo* treatment of the LFS-associated SHH-MB DKFZ-BT084 PDX with adavosertib and VCR had no significant effect on either tumor growth or animal survival (log rank test: *p* > 0.05; Figures 5A and 5B). Median lifespan since tumor detection in each treatment group was respectively: control: 17.5 days, adavosertib: 9 days, vincristine: 20.5 days, combination: 28 days. To evaluate *in vivo* on-target effect of adavosertib phosphorylation of CDK1 was quantified in two other LFS SHH-MB PDX models. No difference in p-CDK1 levels was observed between samples treated with adavosertib and VCR and those in the control group (Figure 5C). This result indicates a lack of on-target activity of adavosertib, possibly because of low blood-brain barrier (BBB) penetration, explaining the lack of efficacy of the drug *in vivo*.

To confirm the *in vivo* dependence on WEE1 activity, tumor formation and growth dynamics of LFS SHH-MB PDX model LFS primary upon stable knock-down of WEE1 by expression of shRNA were evaluated. Out of five mice, three (60%) developed a tumor, whereas in control group, injected with LFS SHH-MB PDX with shSCRAMBLE, all five mice (100%) developed tumor (Fisher’s exact test: *p* = 0.44; Figure 5D). There was a significant difference in survival, as all five mice from the control group died due to tumor development during observation period, while two mice in the WEE1 KD group remained alive (*p* = 0.044; Figure 5E).

### Adavosertib and vincristine do not show toxicity in LFS mouse model

An important filtering criterion of the initial drug screen was genotoxicity, with the aim to find drugs not toxic for DNA damage prone LFS patients. To investigate the possibility of toxicity in LFS patients, adavosertib and VCR were tested in a Trp53^+/−^ LFS mouse model (Trp53^tm1Tyj^/J). During the treatment and observation period, the animals did not show side effects and maintained constant weight (Figure S3A). Survival in the control, single-drug and combination treatment groups, respectively, was 100% (Figure S3B). Moreover, autopsies of the animals showed no macroscopic changes in the organs indicative of tumorigenesis. No abnormalities were found by histological evaluation of brain, kidney, liver, and spleen tissue. No statistical differences in number of γH2AX-positive nuclei in these organs between the control and treatment groups were observed (Figures S3C and S3D). These results indicate low to no genotoxicity or organ-specific toxicities in the Trp53-deficient mouse model upon treatment with VCR, adavosertib, or their combination.

## Discussion

In this study, we evaluated 333 compounds as possible novel treatment options against LFS-associated MB. The results indicated the most effective, non-toxic drug combination to be the WEE1 inhibitor adavosertib plus the vinca alkaloid vincristine. Both compounds did not cause genotoxic effect in *in vitro* and *in vivo* models of LFS, and their combined effect on cancer cell lines was classified as additive. Despite a lack of *in vivo* efficacy of the chosen drug combination, genetic WEE1 KD in an *in vivo* PDX model led to reduction of tumor growth, as well as increased survival of immune-deficient mice, proving WEE1 as a promising target in LFS-associated SHH-MB. Importantly, the mechanism of adavosertib and vincristine combination might not be specific for TP53^mut^ SHH-MB: while TP53^mut^ cancers are sensitive to it, non-mutated cancers might respond as well. The low or absent genotoxicity of adavosertib and VCR indicate the possibility of testing them in other LFS-associated cancer entities in follow-up studies.

We provided evidence that TP53^mut^ SHH-MB tumor growth is dependent on WEE1 activity both in *in vitro* and *in vivo* setting. From a mechanistic point of view, *TP53* mutations and consequent defects of G1/S cell cycle checkpoint control make LFS cells more resistant to chemotherapy.^41^^,^^42^ At the same time, these aberrations make fast-dividing cancer cells dependent on G2/M checkpoint control for completing DNA damage repair before entry into mitosis phase. By targeting WEE1, a kinase controlling G2/M checkpoint transition, adavosertib causes premature entry into mitosis, which exacerbates genomic instability and leads to mitotic catastrophe (Figure 2B).^28^^,^^43^^,^^44^ For this reason, many studies proposed *TP53* mutations as a potential biomarker for adavosertib sensitivity^45^^,^^46^; however, clinical trials in adult patients showed conflicting results.^47^^,^^48^^,^^49^

Currently used therapeutic approaches, composed mostly of conventional chemotherapy, fail to ensure long-term progression-free survival in LFS SHH-MB patients (5-year progression-free survival: 20%).^10^ Vincristine is commonly used as part of the TP53^mut^ SHH-MB treatment regime,^10^^,^^50^^,^^51^ whereas adavosertib is currently being tested in early clinical phase trials in pediatric brain malignancies (NCT02095132).^24^^,^^52^ To date no significant benefits for MB patients treated with WEE1 inhibitor were observed, however, no information is available on TP53^mut^ SHH-MB specifically.

WEE1 seems to be an attractive target in medulloblastoma (MB): a systematic literature review by Keller et al. (2022)^23^ found that inhibitors of WEE1 and other cell cycle kinases are the most promising therapeutics in MB as single agents as well as in combination with chemotherapy and radiotherapy (RT). Finally, a study by Harris et al. using integrated genomic analysis also identified WEE1 as an actionable target in MB.^29^

One of the key factors limiting the use of specific compounds in MB therapy is the BBB. Previous studies have already described BBB penetrance of adavosertib in *in vivo* setting. Pokorny et al. showed that the drug distribution in an orthotopic GBM PDX model was heterogeneous and relatively low compared to adavosertib blood levels (5%).^53^ These findings are consistent with the results of the phase I clinical trial in two adult GBM patients (NCT01849146).^54^ In contrast, pharmacokinetic (PK) analysis in a different cohort of GBM patients indicated good BBB penetration of adavosertib after a single oral dose (median unbound drug tumor-to-plasma concentration ratio: 3.2).^26^^,^^55^ Noteworthy, glioblastomas tend to have a fenestrated vasculature,^56^^,^^57^ whereas SHH-MB are characterized by an intact BBB,^58^^,^^59^^,^^60^ which, in combination with the limited ability of adavosertib to penetrate the BBB, could potentially hinder its use in SHH-MB therapy.

Studies in *in vivo* models of brain tumors show that while adavosertib as a single drug or in combination with chemotherapy inhibits growth of subcutaneously injected brain cancer cells,^29^^,^^43^^,^^44^^,^^61^ its efficacy varies in intracranially injected tumors.^53^^,^^62^^,^^63^^,^^64^ In the studies by Pokorny et al.^53^ as well as Moreira et al.*,*^64^ orthotopic models were less sensitive than heterotopic models, supporting the concept that BBB permeability plays a significant role in adavosertib PK limiting drug concentration at the tumor site. Altered BBB structure in SHH-MB PDX models^65^^,^^66^^,^^67^ makes unambiguous interpretation of these results even more difficult. Our study proved that WEE1 is an attractive target, hence, further efforts should be focused on developing new BBB-penetrant WEE1 inhibitors. Currently a few potential candidates are in clinical development, including ZN-c3 (NCT04158336) and Debio 0123 (NCT03968653),^68^ which in our study showed similar efficacy in TP53^mut^ brain tumor models to adavosertib. Moreover, *in vivo* testing of Debio 0123 in an orthotopic GBM model yielded positive results and indicated its ability to cross BBB.^69^ Other *in vivo* studies are also currently exploring novel drug delivery systems involving nanoparticles^70^^,^^71^ or polymer micelles.^72^^,^^73^^,^^74^ Alternatively, non-invasive techniques such as focused ultrasound with microbubbles, under ongoing clinical evaluation in gliomas, may improve the BBB penetrance of WEE1 inhibitors.^75^ Identification of LFS-specific biomarkers for adavosertib sensitivity could help enhancing its therapeutic potential, however, these efforts may be impeded by the small number of LFS SHH-MB patients.

An important issue in the implementation of new therapies for patients affected by CPS is genotoxicity of therapeutic substances. In LFS patients treated with DNA damaging agents the risk of secondary cancers is highly elevated,^15^^,^^16^^,^^17^ therefore, we examined the ability of the drug hits to cause DNA double-strand breaks and persisting DNA damage. Despite its radio- and chemosensitizing properties, the results of clinical trials did not determine whether adavosertib treatment alone could cause DNA damage.^47^^,^^76^^,^^77^ In this study, extended exposition to adavosertib did not result in increased γH2A.X foci or micronuclei formation in non-cancerous LFS cells. Vincristine as microtubule-associated compound may produce inconclusive results in MNT,^22^ however, previous studies showed that vinca alkaloids do not induce significant amount of DNA damage and cancerogenesis in *in vitro* and *in vivo* LFS models.^20^^,^^21^ Moreover, we did not observe any side effects or organ-specific toxicities in the LFS mouse models upon treatment with adavosertib and vincristine linked to the *Trp53*-deficient background. Based on our findings, we conclude that adavosertib and vincristine can be safely included into treatment regimens of LFS patients.

A potential shortcoming of preclinical studies like the one presented here is the choice of models, which may limit the translatability of the pre-clinical data. Indeed, the number of robust and faithful *in vitro* TP53^mut^ SHH-MB models is very limited. To date, the search for specific and targeted clinical therapies for SHH-MB did not yet render promising results. A study by Pak et al. showed that HDAC inhibitors are promising combination partners in SMO inhibitor-resistant MB tumors,^78^ but these findings have yet to be confirmed by clinical trials, in particular in SHH-MB.^79^ In the study by Endersby et al., CHK1 inhibitors were proven effective in Group 3 MB, but not TP53^mut^ SHH-MB PDX model.^80^ Simovic et al. proved high *in vivo* efficacy of carbon ion RT in LFS SHH-MB, but its efficacy was not improved by combination with PARP inhibitors.^78^ Of note, the clinical value of RT is reflected in our clinical LFS-MB series, indicating that RT prolongs survival.^10^ Regarding therapy in other LFS-associated brain tumors, in Reed et al. a comparative transcriptomics-based precision medicine platform identified STAT1/STAT2 overexpression and ruxolitinib sensitivity in an LFS glioblastoma patient, leading to personalized treatment and disease stabilization.^79^

To find novel and efficient cancer therapies patient-derived models are often used for screening purposes. This approach allows to search for targetable “drivers” relevant for a specific patient or a small patient group. In preclinical studies great importance is given to testing compounds in *in vivo* setting such as genetically engineered animal models and patient-derived xenografts (PDX).^81^^,^^82^^,^^83^ Furthermore, organoid-based *ex vivo* assays are also gaining popularity, as a functional precision medicine approach, in many clinical trials^84^ (NCT03896958, NCT03336931, NCT03133273).

In conclusion, we identified two drug hits, the WEE1 inhibitor adavosertib and the vinca alkaloid vincristine, by a drug screening process and validation, potentially suitable for translation into clinical trials for the treatment of LFS-MB. This combination showed relatively low genotoxicity in LFS non-cancerous cells *in vitro,* and high efficacy in LFS-associated SHH-MB models. The safety profile of this drug combination was confirmed *in vivo* in a genetic LFS mouse model. However, the *in vivo* effectiveness of the selected drug combination was not validated in our study. Based on these findings presented here, a WEE1 inhibitor could be included into treatment regimens of LFS-associated SHH-MB as a combination partner for vincristine, provided the BBB penetration could be improved. Clinical studies are required to test the combination of vincristine and a BBB-penetrant WEE1 inhibitor in LFS SHH-MB, as well as possibly in other LFS-associated tumor entities.

### Limitations of the study

In the study presented here, we focused on the combination of the screening hits vincristine and adavosertib as a potential novel therapeutic approach in LFS-associated SHH-MB. The comprehensive drug library used for screening included mainly drugs either approved or in clinical development. While our initial drug library screening was tested in only two TP53^mut^ brain tumor cell lines, the screen hits were validated in three additional *bona fide* TP53^mut^ SHH-MB PDOs faithfully representing this tumor entity. Another limitation might be the relatively short follow-up time (4 weeks) of the toxicity *in vivo* experiment with the LFS mouse model, potentially not allowing verification of long-term toxicity such as therapy-induced tumorigenesis. Nevertheless, lack of significant increase in DNA damage marker could suggest a low risk of therapy-related secondary tumors. Lastly, the animal models used in this study do not fully recapitulate aspects of LFS patients’ phenotype, e.g., in terms of *TP53* mutation spectrum as well as the BBB structure.

## Resource availability

### Lead contact

Further information and requests for resources should be directed to and will be fulfilled by the lead contact, Anna S. Kolodziejczak (a.kolodziejczak@kitz-heidelberg.de).

### Materials availability

• Requests for further information and resources should be directed to and will be fulfilled by the lead contact.
• This study did not generate new unique reagents.

### Data and code availability

• Immunoblot and microscopy images reported in this paper will be shared by the lead contact upon request.
• This paper does not report original code.
• Any additional information required to reanalyze the data reported in this paper is available from the lead contact upon request.

## Acknowledgments

The study was funded by the Bundesministerium für Bildung und Forschung ADDRess grant (01GM1909A and 01GM1909E to C.P.K., S.M.P., and T.M.). The laboratory of T.G.P.G. acknowledges support from the 10.13039/100018674Barbara and Wilfried Mohr Foundation.

## Author contributions

Conceptualization, T.M., D.T.W.J., K.W.P., C.P.K., and S.M.P.; data curation, formal analysis, investigation, visualization, software, A.S.K. and C.M.; Funding acquisition, T.M., K.W.P., C.P.K., and S.M.P.; methodology: A.S.K., H.P., N.M., R.S., J.B., S.O., P.-C.W., and T.G.P.G.; project administration, A.S.K., T.M., C.M., and M.K.; resources, N.M., K.M., C.H.-M., A.E.D., L.M.K., A.E., and P.-C.W.; supervision, T.M., O.W., M.K., T.G.P.G., and I.O.; validation, F.S., N.J., and R.S.; writing – original draft, A.S.K. and T.M.; writing – review and editing: all coauthors.

## Declaration of interests

O.W. participated in advisory boards of Novartis, BMS, Janssen and receives research grants from BVD, Day One Therapeutics. I.O. receives research grants from PreComb, BVD and Day One Therapeutics.

## STAR★Methods

### Key resources table

**Table:** 

REAGENT or RESOURCE	SOURCE	IDENTIFIER
**Antibodies**		

Phospho-Histone H2A.X (Ser139) (20E3) Rabbit mAb	Cell Signaling Technology	Cat# 9718, RRID:AB_2118009
Polyclonal biotinylated goat anti-rabbit IgG antibody	Dianova	Cat# 111-065-144
α-CDK1 (phospho Y15) [BLR101H]	Abcam	Cat# ab275958
α-Cdk1/Cdk2 (AN21.2)	Santa Cruz Biotechnology	Cat# sc-53219, RRID:AB_2120095
HSP90 (C45G5) Rabbit mAb	Cell Signaling Technology	Cat# 4877, RRID:AB_2233307
Wee 1 (B-11)	Santa Cruz Biotechnology	sc-5285, RRID:AB_628447

**Chemicals, peptides, and recombinant proteins**		

Advanced DMEM	Gibco	Cat# 12491015
Neurobasal medium	Gibco	Cat# 21103049
N2 supplement	Gibco	Cat# 17502001
B27 supplement	Gibco	Cat# 12587001
HEPES buffer	Gibco	Cat# 15630056
GlutaMAX	Gibco	Cat# 35050061
Penicillin-Streptomycin	Gibco	Cat# 15140122
Heparin Solution	Stemcell Technologies	Cat# 07980
epidermal growth factor (EGF) 20 ng/ml	Peprotech	Cat# AF-100-15
fibroblast growth factor (FGF2) 10 ng/ml	Peprotech	Cat# 100-18B
Red Blood Cell Lysis Buffer	Roche	Cat# 11814389001
DMEM - high glucose	Sigma Aldrich	Cat# D5796-500ML
fetal bovine serum (FBS)	Sigma Aldrich	Cat# F7524-500ML
Custom SelleckChem drug library (See Table S1)	SelleckChem	Cat# L2000
TDSU core library	Peterziel et al.^85^	N/A
Hoechst 33342	Thermo Fisher Scientific	Cat# H3570
CellMask™ Deep Red Plasma Membrane Stain	Invitrogen	Cat# C10046
NeuroCult™ NS-A Proliferation Kit	STEMCELL Technologies	Cat# 05751
MEM alpha Medium	Thermo Fisher Scientific	Cat# 12571063
Staurosporine	BIO-CONNECT BV	Cat# 62996-74-1
IVISbrite D-Luciferin Potassium	Revvity	Cat# 122799
TrypLE	Gibco	Cat# 12605010
Roti®-Histofix 4%	Roth	Cat# P087.5
Haematoxylin	Morphisto	Cat# 1193202500
Eosin	Leica	Cat# 3801602E
Alkaline phosphatase streptavidin	VectorLabs	Cat# SA-5100
RED	Dako	Cat# 5005
Haemalaun	Roth	Cat# T 865.3
Pierce Protease inhibitor Mini tablets	Thermo Fisher Scientific	Cat# A32953
PhosSTOP	Roche	Cat# 4906845001
NuPAGE LDS Sample buffer (4x)	Thermo Fisher Scientific	Cat# NP0007
NuPAGE™ Sample Reducing Agent	Thermo Fisher Scientific	Cat# NP0004
Lipofectamine RNAiMAX Transfection Reagent	Invitrogen	Cat# 13778075

**Critical commercial assays**		

CellTiter-Glo® 2.0 Cell Viability Assay	Promega	Cat# G9243
BCA Protein Assay Kit	ThermoFisher Scientific	Cat# 23225
Amersham ECL Prime Western Blotting Detection System	GE Healthcare	Cat# RPN2232SK
Caspase-3 Assay Kit (Fluorometric)	Abcam	Cat# ab39383

**Deposited data**		

ProTrack: Pediatric Brain Tumor	Petralia et al.^84^	http://pbt.cptac-data-view.org/
Tumor Medulloblastoma - Cavalli - 763 - rma_sketch - hugene11t	Cavalli et al.^83^	GSE85217
Tumor Medulloblastoma MAGIC - Northcott - 285 - rma_sketch - hugene11t	Northcott et al.^34^	GSE37382
Normal cerebellum - Roth - 9 - MAS5.0 - u133p2	Roth et al.^33^	GSE3526

**Experimental models: Cell lines**		

Patient-derived xenograft line DKFZ-BT084	Purzner et al.^86^	RRID:CVCL_UK69
Patient-derived xenograft line LFS_primary	Simovic et al.^77^	N/A
Patient-derived xenograft line HS231222	This study	N/A
Patient-derived organoid line B062-008	Meulenbroeks et al., manuscript in preparation	N/A
Patient-derived organoid line B062-13315	Meulenbroeks et al., manuscript in preparation	N/A
Patient-derived organoid line MED113FH	Brabetz et al.^87^	N/A
Cancer cell line CAS-1	Barbieri et al.^88^	RRID:CVCL_1117
Cancer cell line MGBM-1	Moriyama et al.^89^	CVCL_W447
Cancer cell line SF-188	Bodell et al.^90^	RRID:CVCL_6948
Cancer cell line SJ-GBM2	Houghton et al.^91^	RRID:CVCL_M141
Non-cancerous cell line MDAH087	Little et al.^92^	RRID:CVCL_E213

**Experimental models: Organisms/strains**		

NOD.Cg-Prkdc^scid^ Il2rg/SzJ mouse strain	The Jackson Laboratory	JAX: 005557, RRID:IMSR_JAX:005557
129-Trp53^tm1^Tyj/J mouse strain	The Jackson Laboratory	JAX:002080, RRID:IMSR_JAX:002080

**Oligonucleotides**		

ON-TARGETplus Human WEE1 siRNA (SMARTPool)	Dharmacon Reagents	Cat# L-005050-00
ON-TARGETplus Non-targeting Control Pool	Dharmacon Reagents	Cat# D-001810-10-05
α-WEE1 shRNA (CCACCCAGAGTAATAGAACAT)	Chang et al.^93^	N/A

**Recombinant DNA**		

pGreenFire1-ISRE Lentivector	System Biosciences, LLC	Cat# TR016PA-1
pLKO.1 – TRC cloning vector	Addgene	Cat# 10878, RRID:Addgene_10878
shSCRAMBLE vector	Addgene	Cat# 1864, RRID:Addgene_1864

**Software and algorithms**		

ImageJ (version 1.50b)	Wayne Rasband and co., NIH	https://imagej.nih.gov/ij/
Fiji	NIH	https://imagej.net/software/fiji/
CellProfiler version 4.1.3	Broad Institute	https://cellprofiler.org/
Synergy Finder + (version 04.06.2023-R-3.8.2-dev)	University of Helsinki	https://synergyfinder.org/
R version 4.2.2	GNU project	https://www.r-project.org/

**Other**		

Tecan SPARK Multimode Microplate Reader	Tecan	RRID:SCR_021897
ImageXpress Micro 4 High-Content Imaging System	Molecular Devices, LLC	N/A
Beckman Coulter Vi-CELL XR Cell Viability Analyzer	Beckman Coulter, Inc.	RRID:SCR_019664
FACS Aria II Flow Cytometry Cell Sorter	Becton Dickinson	N/A
Multidrop Combi system dispenser	ThermoFisher Scientific	Cat# 17801692
Tecan D3003 Digital Dispenser	Tecan	Cat# F0L56A
CLARIOstarplus Microplate reader	BMG Labtech	N/A
Leica DM3000 LED microscope	Leica	N/A

### Experimental model and study participant details

#### Animals

Animal experiment permits (G-193/21 and G-135/23) were issued by the Regional Council in Karlsruhe. In *in vivo* drug treatment and *in vivo* target dependence experiments female NOD.Cg-Prkdc^scid^Il2rg^tm1Wjl^/SzJ (NSG) mice were used. Intracranial patient-derived xenograft (PDX) tumor cell injections were performed in 6-8-week old animals. Following LFS-associated SHH-MB PDX models were injected: DKFZ-BT084^86^ (relapsed tumor), LFS_primary^78^ (gift from Prof. Aurélie Ernst, treatment-naive) and HS231222 (newly-established model, treatment-naive). After tumor detection mice were randomly assigned into 4 treatment groups (4-5 animals/group) without blinding.

*In vivo* toxicity assessment was performed in Trp53^tm1Tyj^/J (Trp53^+/-^) 129/sv female mice (gift from Pei-Chi Wei). At the age of 6-8-weeks mice were randomly assigned into 4 treatment groups (3-4 animals/group) without blinding.

For both experiments group sizes were calculated using statistical power analysis.

#### Patient-derived organoid (PDO) tumoroid

Patient-Derived Organoid (PDO) tumor cells were established according to a modified protocol from Paassen et al.^94^: in short, fresh tumor material was cut into 1–3 mm^3^ pieces, washed with PDO medium (50% Advanced DMEM (Gibco, cat.no. 12491015), 50% Neurobasal (Gibco, cat.no. 21103049) supplemented with N2 (Gibco, cat.no. 17502001), B27 (Gibco cat.no. 12587001), HEPES buffer (Gibco, cat.no. 15630056), GlutaMAX (Gibco, cat.no. 35050061), Penicillin-Streptomycin (Gibco, cat.no. 15140122), Heparin Solution (Stemcell Technologies, cat.no. 07980), epidermal growth factor (EGF) 20 ng/mL (Peprotech, cat.no. AF-100-15), fibroblast growth factor (FGF2) 10 ng/mL (Peprotech, cat.no. 100-18B)) and spun down at 250 × *G* at 4*°*C. In case of a visible red pellet, 2 mL of Red Blood Cell Lysis Buffer (Roche, cat.no. 11814389001) was added and incubated for 5 minutes at RT. Next, the washing step was repeated and cells were cryopreserved. Cells were cultured as three dimensional (3D) tumoroids in suspension plates in a 37 °C, 5% CO_2_ incubator. PDO medium was refreshed every 3-4 days and cells were split every 2-3 weeks. All PDO models were classified as SHH-MB based on DNA methylation array analysis (Table S6).^95^

#### Established cell lines

For *in vitro* drug screening experiments the following models were used: 4 human glioblastoma multiforme (GBM) cell lines: CAS-1, MGBM-1, SF-188 and SJ-GBM2 (three latter cell lines were kindly provided by Prof. David Jones, DKFZ), SHH-medulloblastoma (MB) cell line UW228-2. All cancerous cell lines were cultured using DMEM - high glucose (cat.no. D5796-500ML, Sigma Aldrich) + 10% heat-inactivated fetal bovine serum (FBS, cat.no. F7524-500ML, Sigma Aldrich) in a 37 °C, 5% CO2 incubator and cells were split every 3-4 days.

Non-cancerous LFS fibroblasts (MDAH087, a gift from Prof. Aurélie Ernst) were using MEM alpha Medium (cat.no. 12571063, Thermo Fisher Scientific) + 10% FBS + 1% GlutaMAX + 1% PenStrep in a 37°C, 5% CO2 incubator. Media was refreshed every 3-4 days and cells were split once a week.

All cell lines were regularly tested for mycoplasma contamination.

#### *In vivo* LFS PDX models

Cancer tumor samples were obtained as part of the ADDRess consortium project (approved by the ethics committee of the Hannover Medical School, application number: 7233) included in the Cancer-Predisposition-Syndrome Registry 01. Establishment of PDX models was described in the Method Details section (Establishing *in vivo* LFS PDX models).

### Method details

#### Drug library

The drug library was constructed of 257 compounds provided by SelleckChem and 76 compounds constituting an internal TDSU core library.^85^ All compounds were stored in form of 10 mM DMSO stock solutions at -80°C. Names of drugs, their targets and classification are listed in Table S1.

#### Single drug screening process

A pilot experiment for *in vitro* drug screening was conducted in 3 biological replicates using 4 human glioblastoma multiforme (GBM) cell lines: CAS-1, MGBM-1, SF-188 and SJ-GBM2 (three latter cell lines were kindly provided by Prof. David Jones, DKFZ) and SHH-MB cell line UW228-2. The cells were seeded in 384-well plate format at the appropriate density (CAS-1: 2500 cells/well, MGBM-1: 1250 cells/well, SF-188: 1500 cells/well, SJ-GBM2: 1500 cells/well, UW228-2: 1500 cells/well). After 24 hours DMSO (final concentration: 0.1%, as a negative control), staurosporine (final concentration: 0.25 μM, as a positive control), benzalkonium chloride (BzCl, final concentration: 100 μM, as a death control), as well as 52 selected drugs in 5 different concentrations (1 nM - 10,000 nM) were added to the cell culture medium. Following 72-hour exposure cell viability was measured using CellTiter-Glo 2.0 Assay (cat.no. G9241, Promega) and the Spark microplate reader (Tecan).

The remaining 281 compounds were screened using the same conditions and concentration range in 3 cell lines representing LFS: SJ-GBM2 (GBM; *TP53* p.R273C), UW228-2 (SHH-MB, *TP53* p.T155N), LFS fibroblasts (non-cancerous control, *TP53*p.R248W). Seeding densities were as follows: SJ-GBM2: 1500 cells/well, UW228-2: 1500 cells/well, LFS fibroblasts: 3000 cells/well. The drug was considered a positive hit, if it fulfilled the following criteria: I) drug sensitivity score 3 (DSS_3_) score^19^ below 10 in non-cancerous cell lines, II) dDSS_3_ (difference between DSS_3_ in cancerous and non-cancerous cell line) above 20 for both cancer cell lines.

To check for false positives the hits after the genotoxicity screen (see a paragraph below) drugs were validated using wider concentration range (0.1 – 10,000 nM) in 3 technical replicates in 3 independent biological replicates in SJ-GBM2, UW228-2, and LFS fibroblasts using the same conditions as in the previous experiment.

DSS_3_ (single drug screen and validation steps) and E_max_ (validation step only) were estimated based on four-parameter logistic curves.

#### Genotoxicity screen in the LFS fibroblasts

To evaluate genotoxicity of compounds, the Micronucleus Test (MNT)^96^^,^^97^ method was used. A non-cancerous cell line, LFS fibroblasts, was exposed to a single drug in C_max_ concentration available from clinical trials as indicated in Table S1 (or, if unavailable, concentration causing 20% decrease of cell viability according to single drug screen results) for 120 h (5 technical replicates in 2 biological repeats). Afterwards the cells were washed once with sterile PBS and incubated with 1:1000 Hoechst 33342 (cat.no. H3570, Thermo Fisher Scientific) and 1:1000 CellMask™ Deep Red Plasma Membrane Stain (cat.no. C10046, Invitrogen) for 30 minutes at 37°C. Next, the cells were visualized using ImageXpress Micro 4 High-Content Imaging System (Molecular Devices, LLC) and 4 images for each well were analyzed with CellProfiler version 4.1.3 (the pipeline described in the supplementary information).

#### Drug combination screen

Drugs were tested in combinations *in vitro* using TP53^mut^ cancer cell lines SJ-GBM2 and UW228-2. Concentration range was estimated based on the results of the single drugs screen, so that it included C_10_-C_max_ relevant for both cell lines. Cell metabolic activity was measured in the same way as in the single drug screen (3 technical replicates x min. 3 biological replicates for each condition).

#### Establishing *in vivo* LFS PDX models

Tumor samples from DADDR patients registered in the KPS registry were obtained according to the protocol posted on the ADDRess project site (https://www.krebs-praedisposition.de/en/cps-research/address/). Material was minced and pipetted thoroughly until obtaining a uniform cell suspension in NeuroCult™ NS-A Proliferation Kit (cat.no. 05751, STEMCELL Technologies). Cells were counted using Vi-CELL XR Analyzer (Beckman Coulter, Inc.) and injected orthotopically into 5 NSG mice (10^6^ cells / 4 μL / mice). Surplus cells were frozen in supplemented NeuroCult medium + 10% DMSO in liquid nitrogen. Tumor growth was monitored with MRI every 6 weeks. Mice showing clinical symptoms were sacrificed and tumor cells were isolated according to procedure described above.

Additionally, tumor cells were labelled with luciferase. Two million cells were incubated in a well of 6-well plate with a lentivirus carrying GFP- and luciferase-expressing vector (pGreenFire1-ISRE Lentivector, System Biosciences, LLC) for 24 hours at 37°C / 5% CO_2_. Transduced cells were washed with PBS and injected into 5 NSG mice according to procedure described above. Tumor growth was monitored with *in vivo* imaging system (IVIS) every 6 weeks (IVISbrite D-Luciferin Potassium, cat. no 122799, Revvity). After tumor isolation GFP-expressing cells were sorted with FACS Aria II Flow Cytometry Cell Sorter (Becton Dickinson) and re-injected into 3 NSG mice. Cells isolated after the second round of PDX expansion were frozen and stored in liquid nitrogen. In order to test the growth dynamics of a newly established PDX model, cells were injected again into 3-5 NSG mice and tumor growth was monitored every 6 weeks with IVIS.

#### Drug treatment assays in PDOs

PDO tumor cells were collected, enzymatically dissociated using TrypLE (Gibco, cat.no. 12605010) and filtered through a 35 μm mesh strainer (Falcon, cat.no. 352235). Cells were resuspended in fresh medium and plated (B062-13315: 1500 cells/well, B062-008/MED113FH: 2500 cells/well in 40 μL) in ultra-low attachment 384-well plates (Corning, cat.no. CLS3830) using the Multidrop Combi system dispenser (ThermoFisher Scientific) and allowed to form 3D spheres in 24h. Tumoroids were treated with vincristine and adavosertib using the Tecan D3003 Digital Dispenser. Cells were treated with DMSO as negative control and with Staurosporine (10 μM) (BIO-CONNECT BV, cat.no. 62996-74-1) as positive control. Metabolic activity was measured 72h after drug treatment by adding 40 μL of CellTiter-Glo 3D cell viability assay (Promega, cat.no. G9683) to each well, shake for 5 minutes using the Multidrop Combi system dispenser and incubated at room temperature and in the dark for an additional 25 minutes. Thereafter, bioluminescence was measured using the CLARIOstarplus (BMG Labtech) plate reader. Results were normalized to DMSO negative controls (100% viability) and Staurosporine positive controls (0% viability).

#### *In vivo* drug testing in LFS SHH-MB PDX models

Effect of adavosertib and vincristine on tumor growth was tested in 3 luciferase-expressing LFS-associated TP53^mut^ SHH-MB PDX models: DKFZ-BT084,^86^ LFS_primary^78^ (gift from Aurélie Ernst) and HS231222. Cells (in number of 1.5∗10^5^ / 4 μL) were injected orthotopically into cerebellum of 6-8-week old female NSG mice (Janvier Labs). Tumor growth was monitored and quantified by weekly bioluminescence measurements. Upon tumor detection the mice were randomly assigned to the treatment groups and treated for 4 weeks according to the following regime: adavosertib per os (p.o.) (60 mg/kg in 0.5% methylcellulose) 5 times/week, vincristine via intraperitoneal injection (i.p.) (0.5 mg/kg in 0.9% NaCl) once weekly. The control group received treatment with solvents only. All animals were monitored for both brain tumor- and drug toxicity-related symptoms and sacrificed when termination criteria were fulfilled.

#### *In vivo* toxicity testing in LFS mouse models

Trp53^tm1Tyj^/J (Trp53^+/-^) 129/sv female mice (gift from Pei-Chi Wei) of age 6-8 week were treated with adavosertib and vincristine according the same protocol as NSG mice injected with PDX models (see *In vivo* drug testing in LFS-associated SHH-MB PDX models method section). 4-week treatment period was followed by 4-week observation period to monitor for drug toxicity-related symptoms. Afterwards the animals were sacrificed and organs (brain, liver, spleen and kidney) were extracted. Tissues were fixed for 48 hours in Roti®-Histofix 4 % (cat.no. P087.5, Roth) followed by dehydration in 50% Ethanol. Formalin-fixed and paraffin-embedded (FFPE) tissue sections were stained with haematoxylin (cat.no. 1193202500, Morphisto) and eosin (cat.no. 3801602E, Leica) for histological assessment. For γH2AX (p-S139) staining 4 μm FFPE tissue sections were cut, deparaffinized and re-hydrated. Following antigen retrieval by treatment with citrate buffer (pH 6.0), slides were sequentially incubated with: monoclonal rabbit anti-γH2AX (p-S139) primary (1:500) (cat.no. 9718, Cell Signaling), polyclonal biotinylated goat anti-rabbit IgG antibody (1:400) (Dianova # 111-065-144) and alkaline phosphatase streptavidin (1:200) (VectorLabs, #SA-5100). RED (Dako #5005) and Haemalaun (Roth # T 865.3) were used as chromogen and counterstaining, respectively. Antibody- and H&E-stained specimens were evaluated by the pathologist (T.G.P.G.). Images were taken with the Leica DM3000 LED microscope (10-fold magnification) and analyzed using Fiji v.2.16.0 (Java 8) and R version 4.2.2 (packages ggplot2 and ggsignif). Post analysis contrast of presented images was increased by 75%.

#### Adavosertib pharmacodynamic marker

SJ-GBM2 and UW228-2 cell lines were treated with adavosertib (100 nM, 300 nM) for 8 and 24 hours. Protein concentration of cell lysates were estimated using the Pierce BCA Protein Assay Kit (cat. no. 23225, ThermoFisher Scientific). For detecting CDK1 phosphorylation the following antibodies were used: α-CDK1 (phospho Y15) [BLR101H] (cat.no. ab275958, Abcam), α-Cdk1/Cdk2 (AN21.2) (cat.no. sc-53219, Santa Cruz Biotechnology, Inc.), α-HSP90 (cat.no. 4877, Cell Signaling Technology). Western blot bands were visualized using Amersham ECL Prime Western Blotting Detection System (cat. no. RPN2232SK, GE Healthcare) and quantified using ImageJ (version 1.50b, Wayne Rasband and co., NIH). Post analysis brightness and contrast of presented images was increased by 50%.

LFS SHH-MB (LFS_primary and HS231222) PDX samples were extracted from mice one hour after treatment administration (see Method section “*In vivo* drug testing in LFS-associated SHH-MB PDX models”). Tumor cells were isolated, mixed with RIPA buffer including protease (Pierce Protease inhibitor Mini tablets, Thermo Fisher Scientific, cat. no. A32953) and phosphatase inhibitors (PhosSTOP, Roche, cat. no. 4906845001) and incubated on ice for 30 minutes with vortexing every 10 minutes. Next, the lysates were spun at 20000 rpm / 4°C for 30 minutes and the supernatant was mixed with Sample buffer (NuPAGE LDS Sample buffer (4x), Thermo Fisher Scientific, cat. no. NP0007) and (NuPAGE™ Sample Reducing Agent, Thermo Fisher Scientific, cat. no. NP0004), then boiled for 10 minutes at 70°C. Prepared samples were used for detecting proteins as described for SJ-GBM2 and UW228-2 cell lines.

#### WEE1 knockdown in TP53^mut^ brain tumor models

SJ-GBM2 and UW228-2 cells were transfected with ON-TARGETplus Human WEE1 siRNA (SMARTPool, cat.no. L-005050-00, Dharmacon Reagents) using Lipofectamine RNAiMAX Transfection Reagent (cat. no. 13778075, Invitrogen). WEE1 knockdown at protein level was confirmed by Western blot (α-WEE1 antibody [B-11], cat.no. sc-5285, Santa Cruz Biotechnology). After 72 hours cell viability was measured as described in Single drug screening method section. Caspase assay was performed 48 hours after treatment according to manufacturer instructions (cat.no. ab39383, Abcam).

LFS_primary PDX cell line was transduced with lentiviral particles (10 particles/cell) containing pLKO.1 – TRC cloning vector (Plasmid #10878, Addgene) with α-WEE1 shRNA (CCACCCAGAGTAATAGAACAT).^93^ After 24 hours cells were injected orthotopically into 5 NSG mice and their growth was monitored as described in Establishing in vivo LFS PDX models method section. LFS_primary cells transduced with shSCRAMBLE vector (Plasmid #1864, Addgene) were used as a negative control.

### Quantification and statistical analysis

#### Reproducibility of *in vitro* drug screening

The method to compare biological replicates was adapted from Niepel et al. 2019.^98^ First, the data were analysed using R-based software (drc, GRmetrics, stats and DSS packages). For each compound parameters derived from curve fitting (IC50 – inhibitory concentration 50, DSS – Drug Sensitivity Score, Iog(GR50) – logarithm of Growth Rate inhibition by 50%, GRmax – maximal Growth Rate inhibition, AOC – Area Over the Curve) as well as standard deviations (SD, Equation 1) and standard errors (SE, Equation 2) were calculated. SD and SE range of respective parameters are presented in Table S7.

Equation 1 $$\sigma =\sqrt{\frac{\sum {\left(x-\bar{x}\right)}^{2}}{\left(n-1\right)}}$$

Equation 2 $$SE=\frac{\sigma \;}{\sqrt{n}}$$

where σ – standard deviation, SE – standard error, x – average value of a calculated parameter, $\bar{x}$ - each value from the dataset, n – sample size, i.e. in Equation 1 number of data points calculated for this dataset (4 cell lines x 52 compounds = 208 data points), in Equation 2 number of biological replicates (3).

All the calculated statistical values for the chosen parameters shown in Table S7 are comparable with average measurement variability obtained by a single scientist in a single research facility presented in Niepel et al. 2019.^98^ These results suggest that our data reproducibility was sufficient to reduce number of biological replicates from n=3 to n=1 for the purpose of medium-throughput drug screen.

#### Genotoxicity testing in LFS fibroblasts

The MNT score for a specific compound was calculated according to a following formula:

$$MNT\;score=\left(\frac{number\;of\;micronuclei}{number\;of\;nuclei}\right)$$

Resulting MNT scores were compared to negative (DMSO) and positive (mitomycin C) control scores using Welch t-test (R version 4.2.2, packages stat, ggplot2). If scores were statistically different from positive control, then the compound was classified as non-genotoxic. If scores were statistically different from negative control, then the compound was classified as genotoxic.

#### Drug combination screen

For established *in vitro* cell lines synergy scores were estimated using SynergyFinder+ (version 04.06.2023-R-3.8.2-dev)^99^ and ranked using the challengeR (version 1.04 in R version 4.2.2).^100^ In analysis of the results from PDO lines Synergy finder+ was used and growth curves were generated using R version 4.2.2 (package drc). Presented synergy scores are averages across 3 biological replicates (each with two technical replicates, from which a mean value was calculated).

#### WEE1 knockdown in TP53^mut^ brain tumor models

Western blot bands were quantified using ImageJ (version 1.50b, Wayne Rasband and co., NIH). The readouts from 3 biological replicates were analyzed using R version 4.2.2 (packages ggplot2, DSS, stats) and presented as mean values with standard deviations.

#### WEE1 expression and adavosertib sensitivity score

The mRNA levels for selected genes were obtained: for pediatric brain tumors from Petralia et al. 2020,^36^ for medulloblastoma from Cavalli et al. 2017^35^ and Northcott et al. 2017^34^ and for human cerebellum from Roth et al. 2006.^33^ Adavosertib sensitivity score was calculated based on expression signature described in Mayoh et al. 2023^39^ as a sum of log_2_ values. The analysis was performed using R version 4.2.2 (packages ggplot2, ggsignif and stats).

#### Animal experiments

At the stage of designing experiments, a power analysis was performed for BT084 and LFS_primary models using R version 4.1.0 (type I error: 5%, type II error: 20%). Survival, IVIS readout and weight was analyzed using R version 4.2.2 (packages stat, ggplot2, ggsignif, survival, survminer and stats). Images were analyzed using Fiji v.2.16.0 (Java 8) and R version 4.2.2 (packages ggplot2 and ggsignif).

## Supplemental information

Document S1. Figures S1–S3, Tables S2–S7, and Data S1–S3

Table S1. Excel file containing additional data too large to fit in a PDF, related to Figure 1B
