# Supplementary material for: Preclinical drug screen identifies WEE1 inhibitor and vinca alkaloid as a combination treatment concept for Li-Fraumeni syndrome medulloblastoma
Source: iScience. 2025 Dec 29;29(2):114564. doi: 10.1016/j.isci.2025.114564 (PMC12828543; doi:10.1016/j.isci.2025.114564)

iScience, Volume *29*

Supplemental information

Preclinical drug screen identifies WEE1 inhibitor and vinca alkaloid as a combination treatment concept for Li-Fraumeni syndrome medulloblastoma

Anna S. Kolodziejczak, Florian Selt, Heike Peterziel, Nora Jamaladdin, Norman Mack, Kendra Maaß, Chris Meulenbroeks, Romain Sigaud, Christel Herold-Mende, Ahmed El Damaty, Jürgen Burhenne, Shunya Ohmura, Tim Holland-Letz, Lena M. Kutscher, Aurélie Ernst, Pei-Chi Wei, Thomas G.P. Grünewald, Ina Oehme, Marcel Kool, David T.W. Jones, Kristian W. Pajtler, Christian P. Kratz, Stefan M. Pfister, Olaf Witt, and Till Milde

Fig. S1. Single drug screen in Li-Fraumeni Syndrome (LFS) *in vitro* cell line models (related to Figure 1):
A) DSS heatmap of single drug screen hits ("Single drug screen in LFS fibroblasts" step in Figure 1A) in established *in vitro* cell lines modelling LFS (DSS_3 – drug sensitivity score 3)
B) Micronucleus assay (MNT) scores of microtubule-associated compounds compared to DMSO (negative control) and mitomycin C (MMC, positive control): horizontal lines: MMC – median MNT score for the positive control (MMC), DMSO – median MNT score for the negative control (DMSO), vertical lines: median MNT scores with 95% confidence interval for tested compounds (see also Table S3)
C) Dose-response curves of validated non-genotoxic drug hits in established *in vitro* cell lines (LFS – Li-Fraumeni Syndrome): data are represented as mean ± SEM (literature sources of C_{max} values are indicated in Table S1)
D) Dose-response curves of adavosertib and BAY-1895344 in UW228-2 and LFS fibroblasts

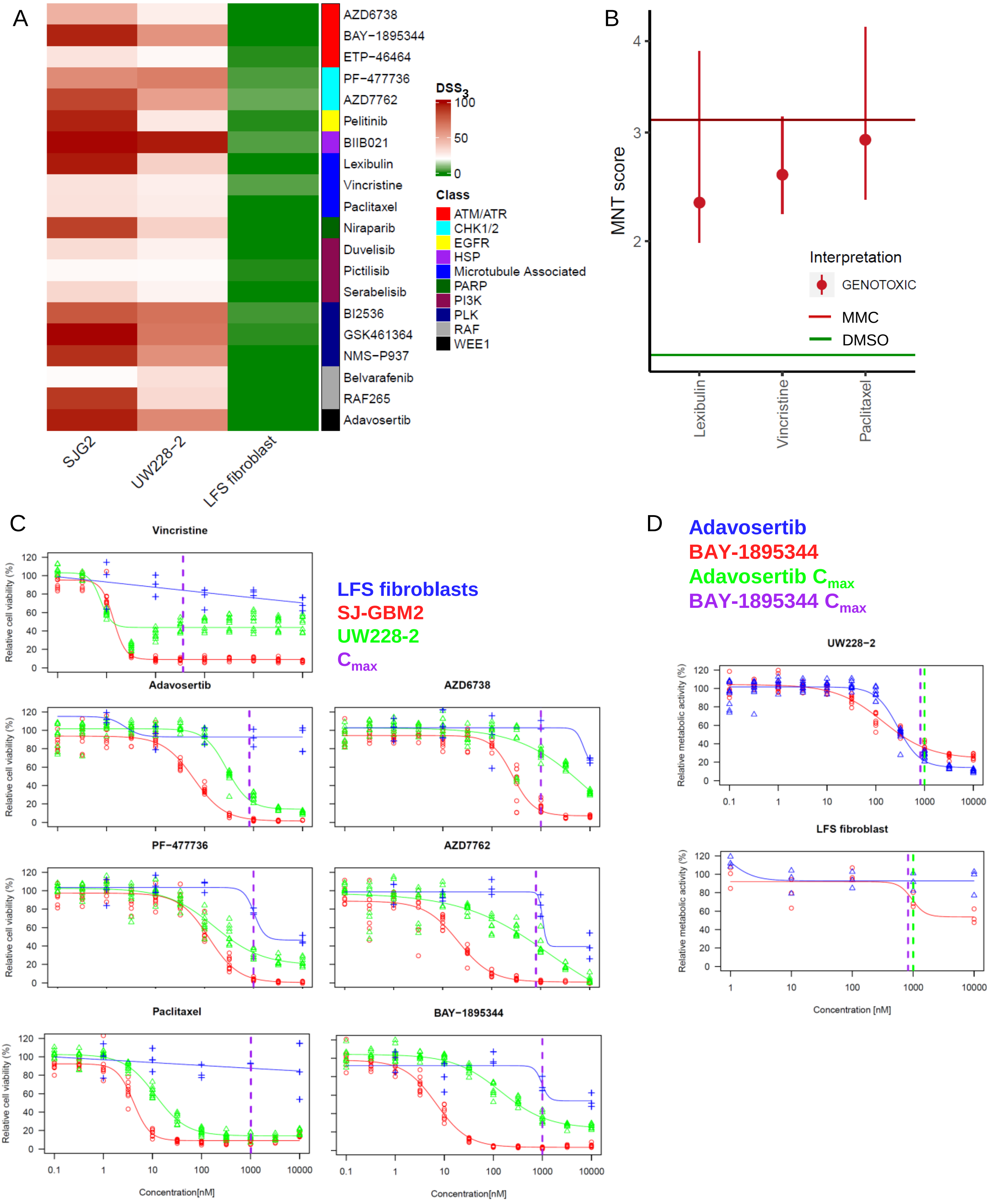

Fig. S2. Validation of drug screen results in SHH-medulloblastoma (SHH-MB) models and clinical patient data:

A) WEE1 protein knockdown in *in vitro* $TP53^{mut}$ brain cancer cell lines shown in Figures 2D-E

B) Highest synergy scores estimated for adavosertib and vincristine combination in *in vitro* SHH-MB patient derived organoid cultures: scores from -10 to 10 are interpreted as additive, scores higher than 10 as synergistic (related to Figure 3A)

C) Adavosertib sensitivity score in $TP53^{wt}$ and $TP53^{mut}$ medulloblastoma (MB) tumors (Petralia et al. (n=22)[S1]); student's t-test was used for statistical analyses, data are represented as mean ± SEM (n.s. – not significant) (related to Figure 4)

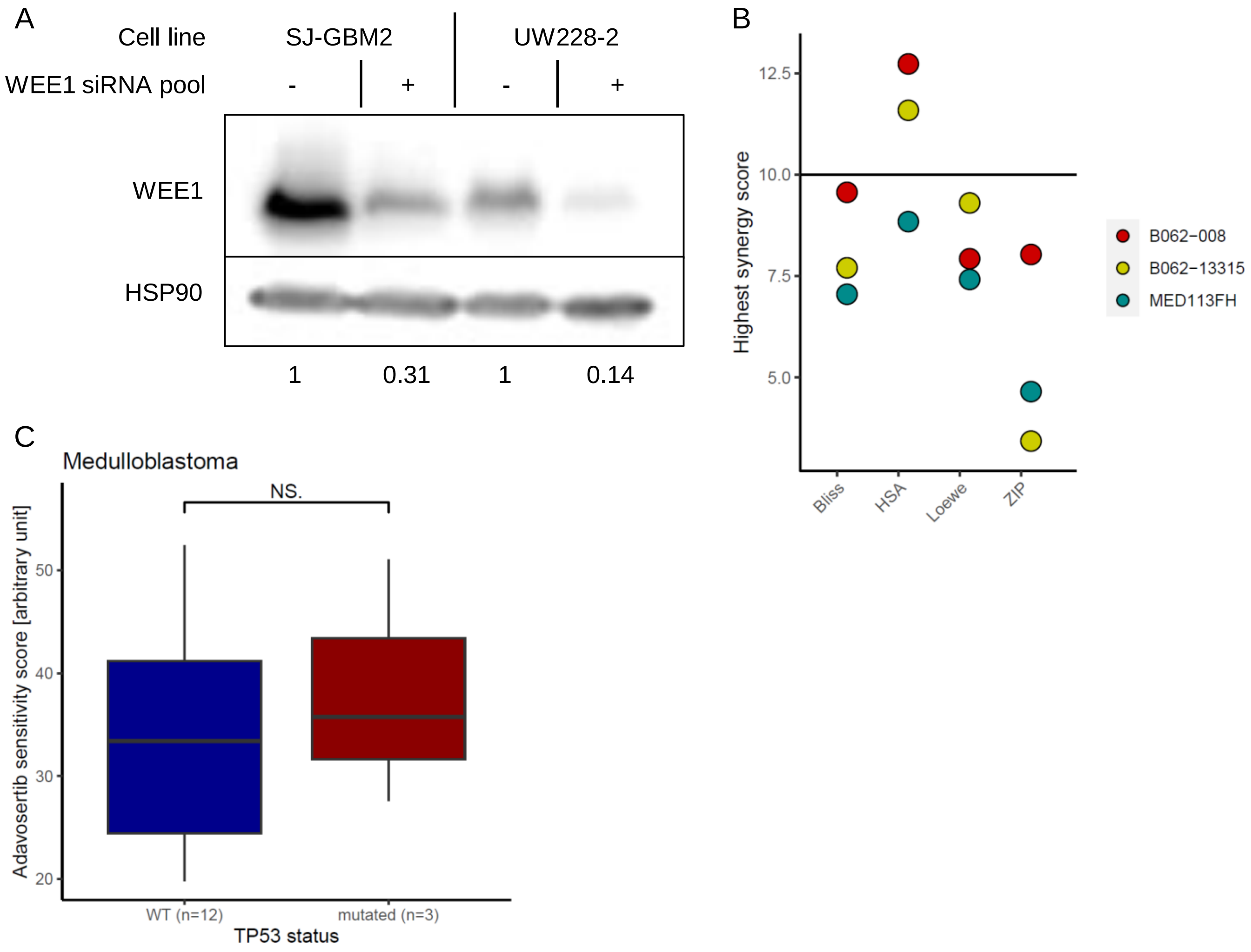

Fig. S3. *In vivo* validation of adavosertib and vincristine (VCR) in Li-Fraumeni Syndrome (LFS) SHH-medulloblastoma (MB) models:

A) LFS mouse model: weight of animals treated with adavosertib and VCR

B) Toxicity testing of adavosertib and vincristine combination Li-Fraumeni Syndrome (LFS) mouse model: mouse survival during treatment and observation period

C) γH2AX immunostaining of Li-Fraumeni Syndrome (LFS) mouse brain, kidneys, livers and spleen treated *in vivo* with adavosertib and vincristine (alkaline phosphatase, magenta) related to Figure S3D

D) Number of γH2AX-positive nuclei and total γH2AX-positive area per field in LFS mouse tissues treated *in vivo* with adavosertib and VCR: the bar plot displays the average value per field across all organs, the individual points represent the value per field for each organ; student's t-test was used for statistical analyses (n.s. – not significant) (see also Figure S3C).

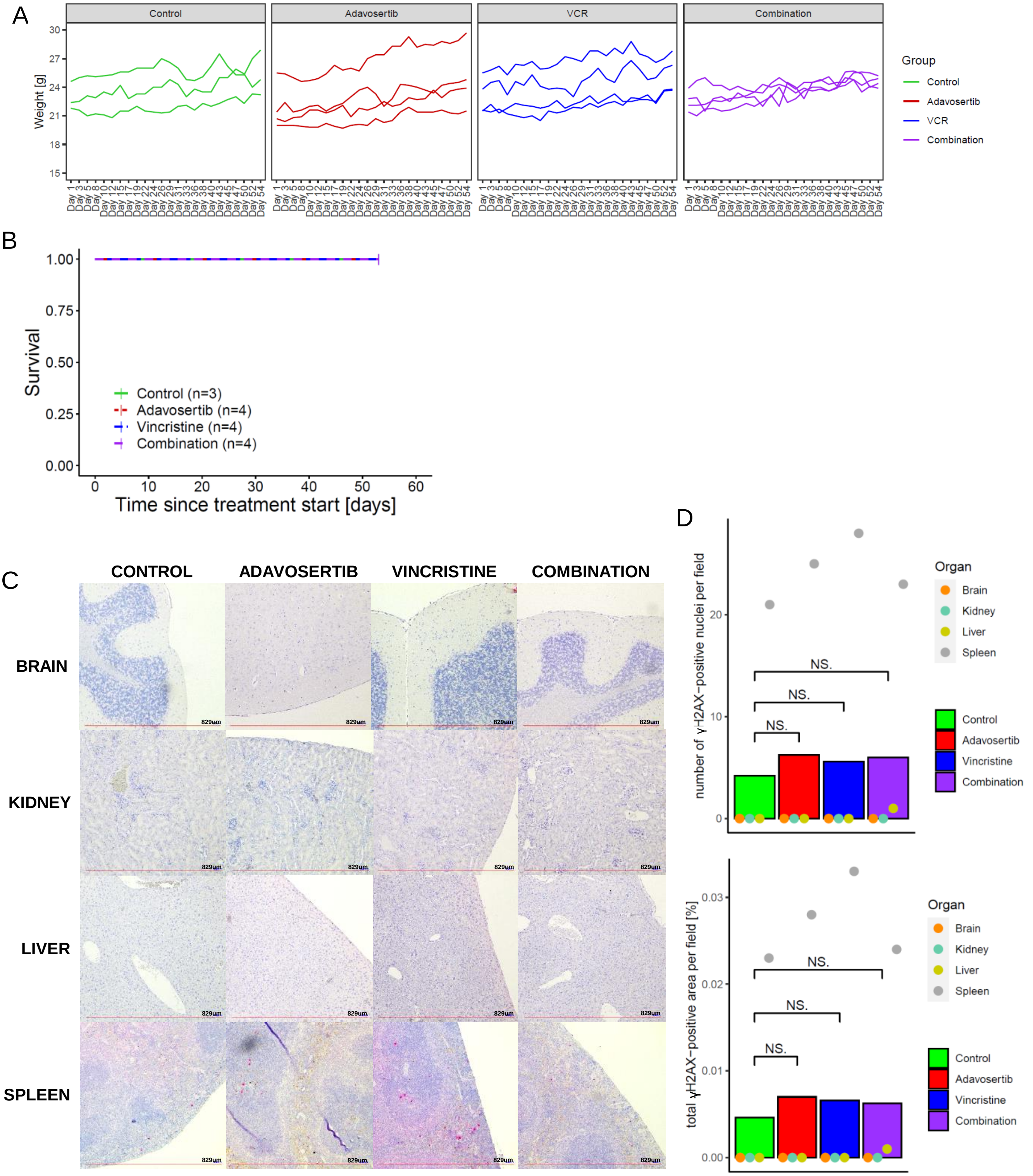

Step	Models used	Criteria for positive selection	Goal
Single drug screen in $TP53^{mut}$ brain cancer cell lines	SJ-GBM2, UW228-2	$DSS_3 \geq 20$ in both models	Selecting compounds effective in $TP53^{mut}$ brain cancer cell lines
Single drug screen in LFS fibroblast	LFS fibroblasts	$DSS_3 \leq 10$ in LFS fibroblasts $dDSS_3 \geq 20$ in both cancer cell lines	Selecting compounds non-toxic for LFS non-malignant tissues
Genotoxic screen in LFS fibroblast	LFS fibroblasts	MNT scores significantly different from the positive control (MMC)	Selecting compounds that do not cause DSBs in LFS non-malignant tissues
Validation in established cell lines	SJ-GBM2, UW228-2, LFS fibroblasts	E_{max} > 50% in cancer cell lines	Selecting compounds cytotoxic for cancer cell lines
Combination screen in cancer cell lines	SJ-GBM2, UW228-2	Most effective combination according to consensus ranking<br>Clinical relevance*	Selecting the most effective drug combination
Validation in $TP53^{mut}$ SHH-MB patient-derived organoids	BT062-008, BT062-13315 MED113FH	N/A	Validation of previous results in patient-derived organoids

Table S2. Models and criteria used in the drug screening pipeline in Li-Fraumeni Syndrome (LFS)-associated models (related to Figure 1A): SHH-MB – SHH-activated medulloblastoma, DSS_3 – Drug Sensitivity Score 3, $dDSS_3$ – difference in DSS_3 between a cancer cell line and a non-malignant control (LFS fibroblasts), MNT – micronucleus test, MMC – mitomycin C, E_{max} – maximal effect, N/A – not applicable;

*** - clinical relevance was evaluated for 2 compounds (adavosertib and BAY-1895344) based on number of clinical trials registered until 26.05.2023, including the trials involving pediatric patients, as well as reported blood-tumor barrier penetration – see details in the result section.**

LN	Name	Target	MNT_median	95%CI lower	95%CI upper	t.test DMSO	t.test MMC	Interpretation
1	AZD6738	ATM/ATR	1,33	1,14	1,98	0,59	0,00	NON-GENOTOXIC
2	BAY 1895344 (BAY-1895344)	ATM/ATR	1,10	0,95	1,68	0,67	0,00	NON-GENOTOXIC
3	ETP-46464	ATM/ATR	0,96	0,78	1,80	0,67	0,00	NON-GENOTOXIC
4	PF-477736	CHK	1,09	0,97	2,00	0,82	0,00	NON-GENOTOXIC
5	AZD7762	CHK	1,15	0,98	1,83	0,96	0,00	NON-GENOTOXIC
6	Pelitinib (EKB-569)	EGFR	0,68	0,61	1,10	0,01	0,00	NON-GENOTOXIC
7	BIIB021	HSP (e.g. HSP90)	3,64	2,34	5,89	0,00	0,33	GENOTOXIC
8	Lexibulin (CYT997)	Microtubule Associated	2,33	1,99	3,88	0,00	0,82	FALSE POSITIVE
9	Niraparib (MK-4827)	PARP	1,76	1,43	3,95	0,05	0,59	GENOTOXIC
10	Duvelisib (IPI-145, INK1197)	PI3K	0,96	0,88	1,45	0,25	0,00	NON-GENOTOXIC
11	Pictilisib (GDC-0941)	PI3K	1,07	0,98	1,41	0,28	0,00	NON-GENOTOXIC
12	Serabelisib (INK-1117,MLN-1117,TAK-117)	PI3K	1,12	1,02	1,69	0,79	0,00	NON-GENOTOXIC
13	BI 2536	PLK	2,10	1,74	3,24	0,01	0,26	GENOTOXIC
14	GSK461364	PLK	2,14	1,80	3,42	0,01	0,39	GENOTOXIC
15	NMS-P937 (NMS1286937)	PLK	2,77	2,19	3,67	0,00	0,79	GENOTOXIC
16	Belvarafenib(GDC5573, HM95573, RG6185)	Raf	3,67	3,03	4,88	0,00	0,13	GENOTOXIC
17	RAF265 (CHIR-265)	Raf	0,89	0,76	1,53	0,28	0,00	NON-GENOTOXIC
18	Adavosertib （MK-1775）	Wee1	1,44	1,34	1,99	0,29	0,00	NON-GENOTOXIC
19	Vincristine	Microtubule Associated	2,59	2,23	3,16	0,00	0,38	FALSE POSITIVE
20	Paclitaxel	Microtubule Associated	2,93	2,36	4,17	0,00	0,73	FALSE POSITIVE

Table S3. Micronucleus assay (MNT) scores in Li-Fraumeni Syndrome (LFS) non-cancerous fibroblast cell line (related to Figure 1D and S1B): results are presented as medians with 95% confidence interval (CI) and compared with negative (DMSO) and positive control (mitomycin C, MMC) using Student's t-test – p-values > 0.05 in comparison with DMSO (t.test DMSO) or p-values < 0.05 in comparison with MMC (t.test MMC) were interpreted as non-genotoxic. Since micronucleus assay gives false positive results for microtubule-associated compounds, the results for these compounds are classified as „false positive".

ID	Combination	ZIP_synergy_score	HSA_synergy_score	Loewe_synergy_score	Bliss_synergy_score	Interpretation	Consensus ranking value
1	Adavosertib-Vincristine	0,776892865	4,389460206	1,307607889	-0,066146931	ADDITIVE	1,875
2	BAY-1895344-Vincristine	0,011200053	4,694011034	2,163712361	-0,537115514	ADDITIVE	2,125
3	Adavosertib-Vinblastine	0,753832266	3,078722741	0,0208686	0,605405637	ADDITIVE	3,875
4	AZD6738-Vincristine	-0,897392948	3,622831847	0,778135482	-1,960741936	ADDITIVE	4,000
5	BAY-1895344-Vinblastine	-1,175923111	3,081506967	0,401369093	-1,369929718	ADDITIVE	5,000
6	AZD7762-Vincristine	-2,968740006	2,143800945	-0,493747468	-3,243171681	ADDITIVE	5,750
7	AZD6738-Vinblastine	-1,186484932	2,801519351	0,012181948	-1,920378436	ADDITIVE	7,000
8	AZD7762-Vinblastine	-2,897386561	1,379789644	-0,983181498	-3,789404465	ADDITIVE	7,250
9	PF-477736-Vincristine	-1,661931381	2,973586322	0,155513545	-2,447444372	ADDITIVE	8,125
10	PF-477736-Vinblastine	-2,057318944	1,550105428	-1,139486455	-4,164771188	ADDITIVE	10,000

Table S4. Results of drug combination screen in cell lines modelling Li-Fraumeni Syndrome (LFS)-associated brain tumors (related to Figures 2A): synergy scores were calculated using Synergy Finder + and ranked using the challengeR package (R version 4.2.2). Scores between -10 and 10 were interpreted as additive interaction.

PDO model	ZIP_synergy	HSA_synergy	Loewe_synergy	Bliss_synergy	ic50_adavosertib	ic50_vincristine	DSS_adavosertib	DSS_vicristine
B062-008	-1,06	2,52	1,61	-1,09	215,26	7,24	8,36	22,69
MED113FH	-2,81	-0,16	-1,07	-2,94	166,90	11,91	9,53	16,96
B062-13315	-5,43	-0,43	-1,32	-6,35	117,39	1,38	17,70	54,34

Table S5. Results of drug validation in in vitro Li-Fraumeni Syndrome (LFS)-associated SHH-medulloblastoma (MB) patient-derived organoid (PDO) models (related to Figure 2D and S2A): synergy scores were calculated using Synergy Finder +, IC_{50} and drug sensitivity scores (DSS) were calculated using R packages drc and DSS, respectively.

PDO Model	Class	Class Score	Outcome
MED113FH	Medulloblastoma, SHH-activated	0.96	MB_SHH
B062_008	Medulloblastoma, SHH-activated	0.99	MB_SHH
B062_13315	Medulloblastoma, SHH-activated	0.98	MB_SHH

Table S6. Li-Fraumeni Syndrome (LFS)-associated SHH-medulloblastoma (MB) patient-derived organoid (PDO) models classification based on Heidelberg Epignostix CNS Tumor Classifier V12.8[S1]: each class produces a confidence score between 0 and 1, and a tumor type is considered positively identified when the methylation class score is greater than 0.9.

	Standard deviation		Standard error		
**Parameter**	**IC50**	**DSS**	**log(GR50)**	**GRmax**	**AOC**
Upper whisker	1,7	1,7	0,5	0,1	0,1
3rd quartile	0,7	0,4	0,2	0,1	0,0
Median	0,4	0,1	0,1	0,0	0,0
1st quartile	0,2	0,0	0,1	0,0	0,0
Lower whisker	0,0	0,0	0,0	0,0	0,0
# of data points	208,0	208,0	208,0	208,0	208,0

Table S7. Parameters of single drug screen reproducibility in $TP53^{mut}$ brain tumor models (related to Method „Reproducibility of in vitro drug screening")

Data S1. Cell Profiler (version 4.1.3) pipeline used in micronucleus assay analysis (related to Figures 2D and S1B, Table S3)

```
CellProfiler Pipeline:
Version:5
DateRevision:413
GitHash:
ModuleCount:10
HasImagePlaneDetails:False
Images:
    Filter images?:Custom
    Select the rule criteria:or (file does contain "_w1")
Metadata:
    Extract metadata?:Yes
    Metadata data type:Text
    Metadata types:
    Extraction method count:1
    Metadata extraction method:Extract from file/folder names
    Metadata source:File name
    Extract metadata from:All images
    Select the filtering criteria:and (file does contain "")
    Metadata file location:Elsewhere...|
    Match file and image metadata:[]
    Use case insensitive matching?:No
    Metadata file name:None
    Does cached metadata exist?:No
NamesAndTypes:
    Assign a name to:Images matching rules
    Select the image type:Grayscale image
    Name to assign these images:DNA
    Match metadata:[]
    Image set matching method:Order
    Set intensity range from:Image metadata
    Assignments count:1
    Single images count:0
    Maximum intensity:255.0
    Process as 3D?:No
    Relative pixel spacing in X:1.0
    Relative pixel spacing in Y:1.0
    Relative pixel spacing in Z:1.0
    Select the rule criteria:and (file does contain "_w1")
    Name to assign these images:DNA
    Name to assign these objects:Cell
    Select the image type:Grayscale image
    Set intensity range from:Image metadata
    Maximum intensity:255.0
Groups:
    Do you want to group your images?:No
    grouping metadata count:1
    Metadata category:None
EnhanceOrSuppressFeatures:
    Select the input image:DNA
    Name the output image:Supress_DNA
    Select the operation:Suppress
    Feature size:3
    Feature type:Speckles
    Range of hole sizes:1,10
    Smoothing scale:2.0
    Shear angle:0.0
    Decay:0.95
    Enhancement method:Tubeness
    Speed and accuracy:Fast
    Rescale result image:No
IdentifyPrimaryObjects:
    Select the input image:Supress_DNA
    Name the primary objects to be identified:Nucleus
    Typical diameter of objects, in pixel units (Min,Max):50,200
    Discard objects outside the diameter range?:Yes
    Discard objects touching the border of the image?:Yes
    Method to distinguish clumped objects:Shape
    Method to draw dividing lines between clumped objects:Shape
    Size of smoothing filter:10
    Suppress local maxima that are closer than this minimum allowed distance:7.0
    Speed up by using lower-resolution image to find local maxima?:Yes
    Fill holes in identified objects?:After both thresholding and declumping
    Automatically calculate size of smoothing filter for declumping?:Yes
    Automatically calculate minimum allowed distance between local maxima?:Yes
    Handling of objects if excessive number of objects identified:Continue
    Maximum number of objects:500
    Display accepted local maxima?:No
    Select maxima color:Blue
    Use advanced settings?:Yes
    Threshold setting version:12
    Threshold strategy:Global
    Thresholding method:Minimum Cross-Entropy
    Threshold smoothing scale:5
    Threshold correction factor:1.0
    Lower and upper bounds on threshold:0.0,1.0
    Manual threshold:0.0
    Select the measurement to threshold with:None
    Two-class or three-class thresholding?:Two classes
    Log transform before thresholding?:No
    Assign pixels in the middle intensity class to the foreground or the background?:Foreground
    Size of adaptive window:50
    Lower outlier fraction:0.05
    Upper outlier fraction:0.05
    Averaging method:Mean
    Variance method:Standard deviation
    # of deviations:2.0
    Thresholding method:Minimum Cross-Entropy
IdentifyPrimaryObjects:
    Select the input image:Supress_DNA
    Name the primary objects to be identified:Micronuclei
    Typical diameter of objects, in pixel units (Min,Max):10,50
    Discard objects outside the diameter range?:Yes
    Discard objects touching the border of the image?:Yes
    Method to distinguish clumped objects:Shape
    Method to draw dividing lines between clumped objects:Shape
    Size of smoothing filter:10
    Suppress local maxima that are closer than this minimum allowed distance:7.0
    Speed up by using lower-resolution image to find local maxima?:Yes
    Fill holes in identified objects?:After both thresholding and declumping
    Automatically calculate size of smoothing filter for declumping?:Yes
    Automatically calculate minimum allowed distance between local maxima?:Yes
    Handling of objects if excessive number of objects identified:Continue
    Maximum number of objects:500
    Display accepted local maxima?:No
    Select maxima color:Blue
    Use advanced settings?:Yes
    Threshold setting version:12
    Threshold strategy:Global
    Thresholding method:Minimum Cross-Entropy
    Threshold smoothing scale:5
    Threshold correction factor:1.0
    Lower and upper bounds on threshold:0.0,1.0
    Manual threshold:0.0
    Select the measurement to threshold with:None
    Two-class or three-class thresholding?:Two classes
    Log transform before thresholding?:No
    Assign pixels in the middle intensity class to the foreground or the background?:Foreground
    Size of adaptive window:50
    Lower outlier fraction:0.05
    Upper outlier fraction:0.05
    Averaging method:Mean
    Variance method:Standard deviation
    # of deviations:2.0
    Thresholding method:Minimum Cross-Entropy
MaskObjects:
    Select objects to be masked:Micronuclei
    Name the masked objects:Micronuclei2
    Mask using a region defined by other objects or by binary image?:Objects
    Select the masking object:Nucleus
    Select the masking image:None
    Handling of objects that are partially masked:Remove
    Fraction of object that must overlap:0.5
    Numbering of resulting objects:Renumber
    Invert the mask?:Yes
CalculateMath:
    Name the output measurement:MNT
    Operation:Divide
    Select the numerator measurement type:Image
    Select the numerator objects:Micronuclei2
    Select the numerator measurement:Count_Micronuclei2
    Multiply the above operand by:1.0
    Raise the power of above operand by:1.0
    Select the denominator measurement type:Image
    Select the denominator objects:Nucleus
    Select the denominator measurement:Count_Nucleus
    Multiply the above operand by:1.0
    Raise the power of above operand by:1.0
    Take log10 of result?:No
    Multiply the result by:1.0
    Raise the power of result by:1.0
    Add to the result:0.0
    How should the output value be rounded?:Rounded to a specified number of decimal places
    Enter how many decimal places the value should be rounded to:6
    Constrain the result to a lower bound?:No
    Enter the lower bound:0.0
    Constrain the result to an upper bound?:No
    Enter the upper bound:1.0
ExportToSpreadsheet:
    Select the column delimiter:Tab
    Add image metadata columns to your object data file?:No
    Add image file and folder names to your object data file?:No
    Select the measurements to export:Yes
    Calculate the per-image mean values for object measurements?:No
    Calculate the per-image median values for object measurements?:No
    Calculate the per-image standard deviation values for object measurements?:No
    Output file location:Default Output Folder|
    Create a GenePattern GCT file?:No
    Select source of sample row name:Metadata
    Select the image to use as the identifier:None
    Select the metadata to use as the identifier:None
    Export all measurement types?:Yes
    Press button to select measurements:Image|Math_MNT
    Representation of Nan/Inf:NaN
    Add a prefix to file names?:Yes
    Filename prefix:211130_
    Overwrite existing files without warning?:No
    Data to export:Do not use
    Combine these object measurements with those of the previous object?:No
    File name:DATA.csv
    Use the object name for the file name?:Yes
```

References:

S1. Petralia F, Tignor N, Reva B, et al. Integrated Proteogenomic Characterization across Major Histological Types of Pediatric Brain Cancer. Cell. Dec 23 2020;183(7):1962-1985.e31. doi:10.1016/j.cell.2020.10.044

S2. Capper, D., Jones, D.T.W., Sill, M., Hovestadt, V., Schrimpf, D., Sturm, D., Koelsche, C., Sahm, F., Chavez, L., Reuss, D.E., et al. (2018). DNA methylation-based classification of central nervous system tumours. Nature 555, 469-474. 10.1038/nature26000.

Data S2. Unedited, uncropped Western Blot images and the molecular weight standards, related to Figures 2C, 5C and S2A (continued on the next page)

Figure 2C

HSP90 (pCDK1 membrane)

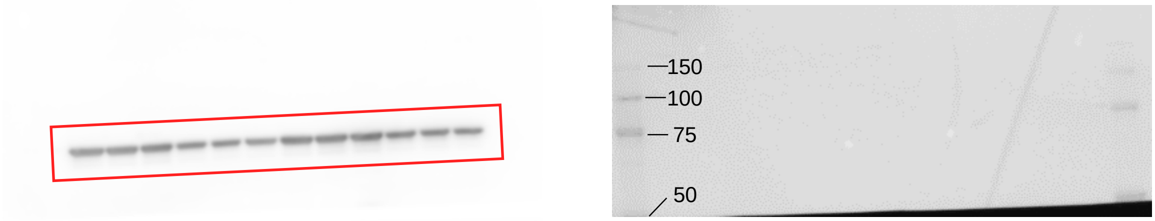

pCDK1

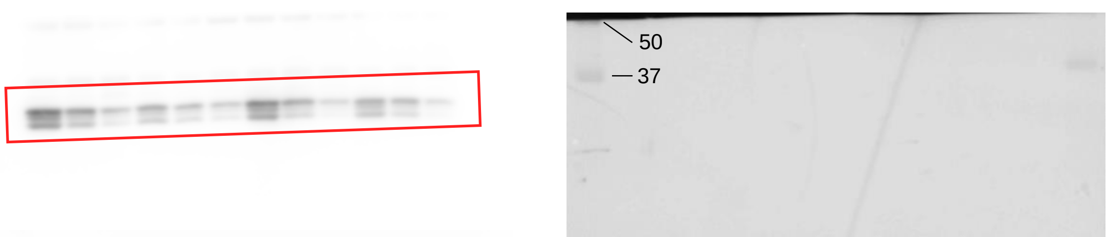

HSP90 (CDK1 membrane)

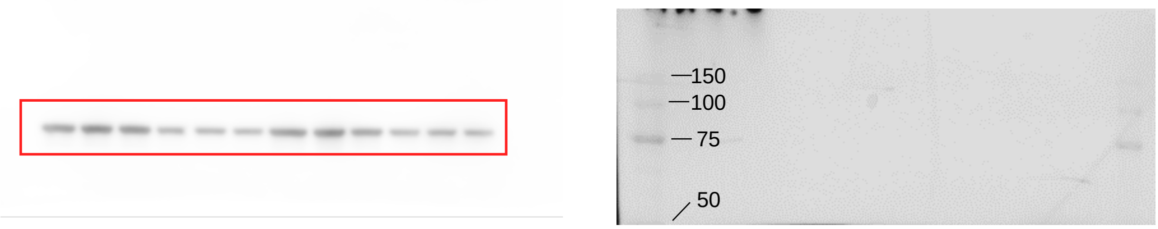

CDK1

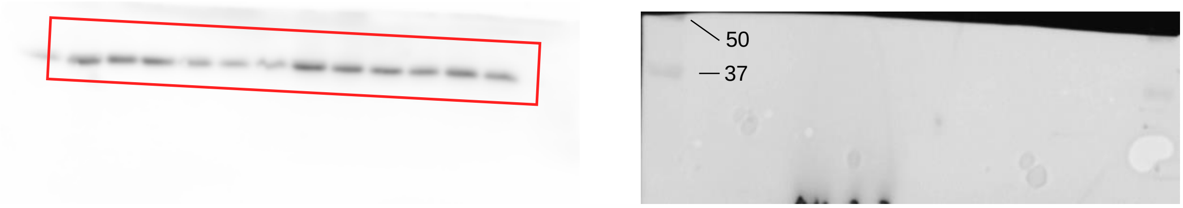

Figure S2A

WEE1

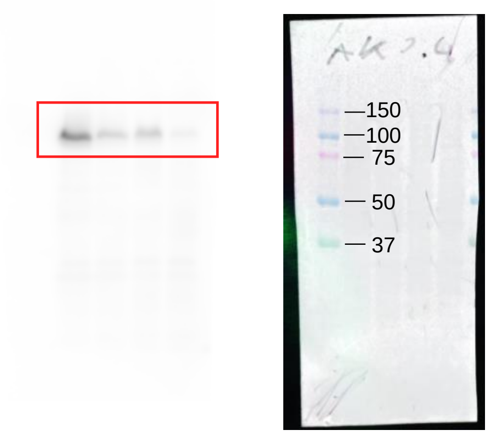

HSP90

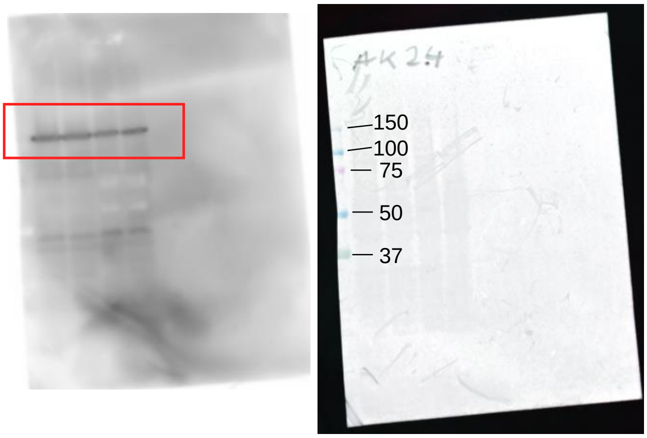

Figure 5C

HS231222

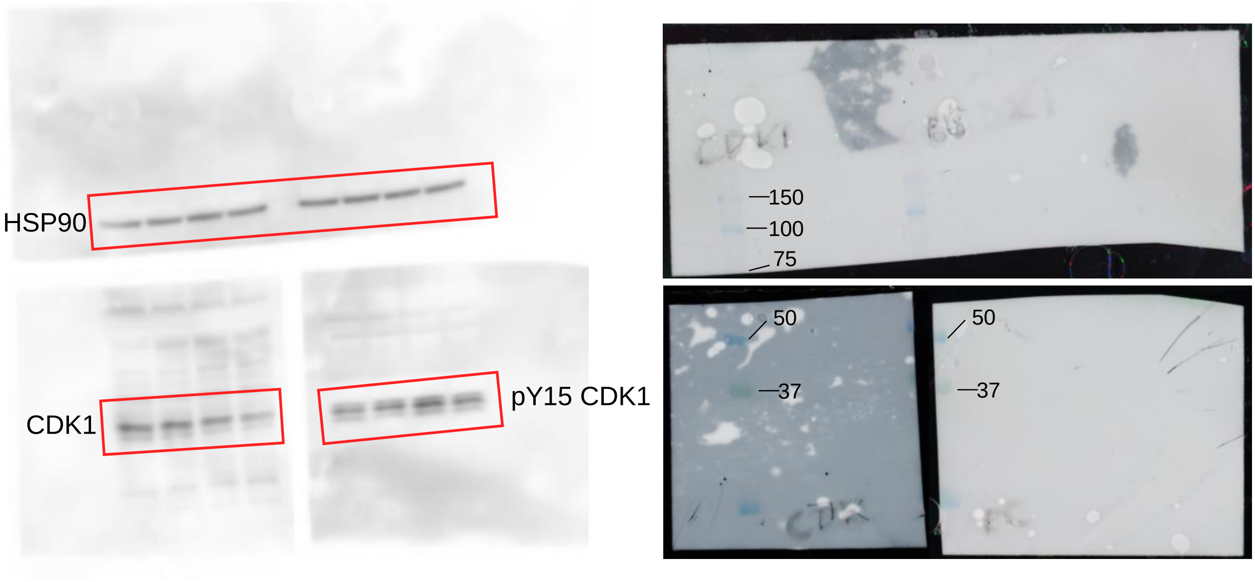

LFS primary

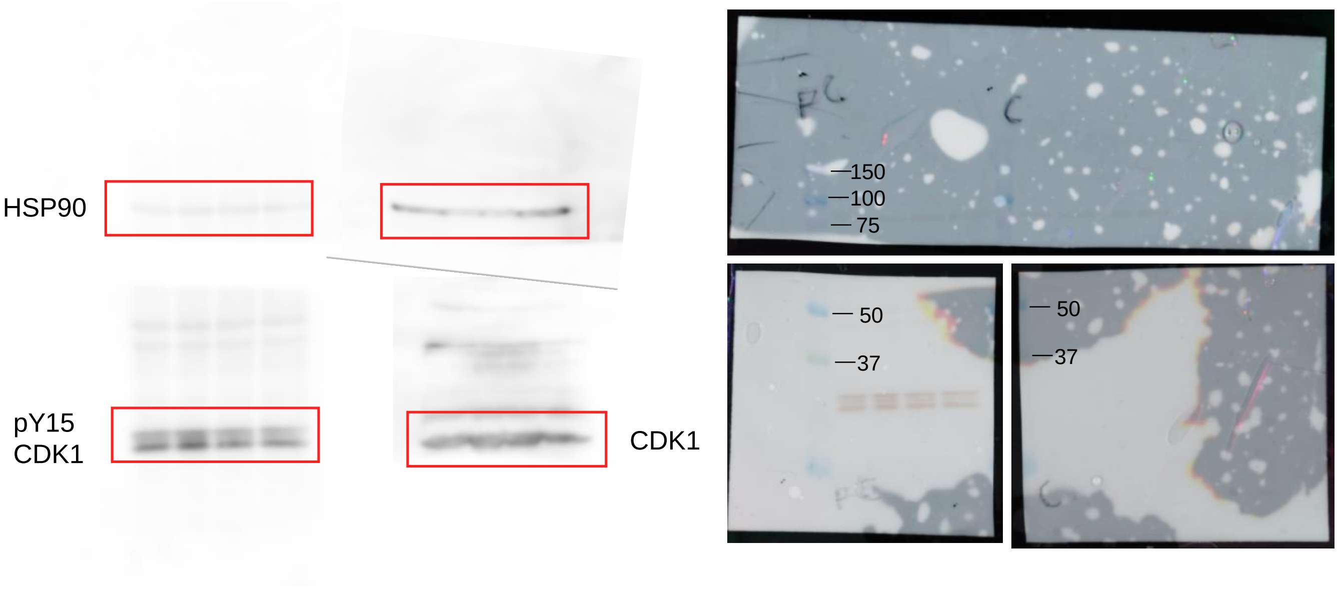

Data S3. Unedited, uncropped microscopy images related to Figure 1D

DMSO

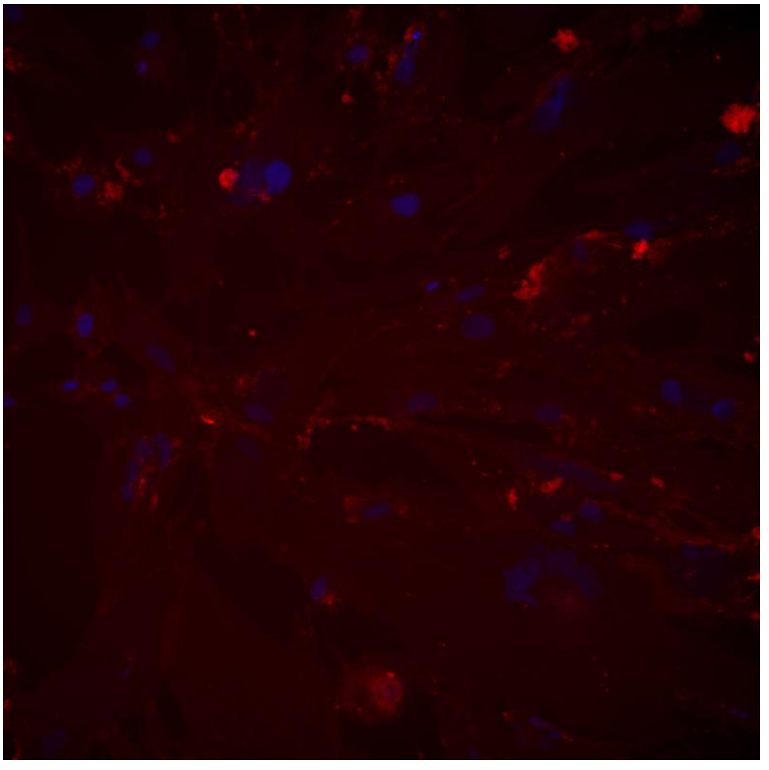

Mitomycin C

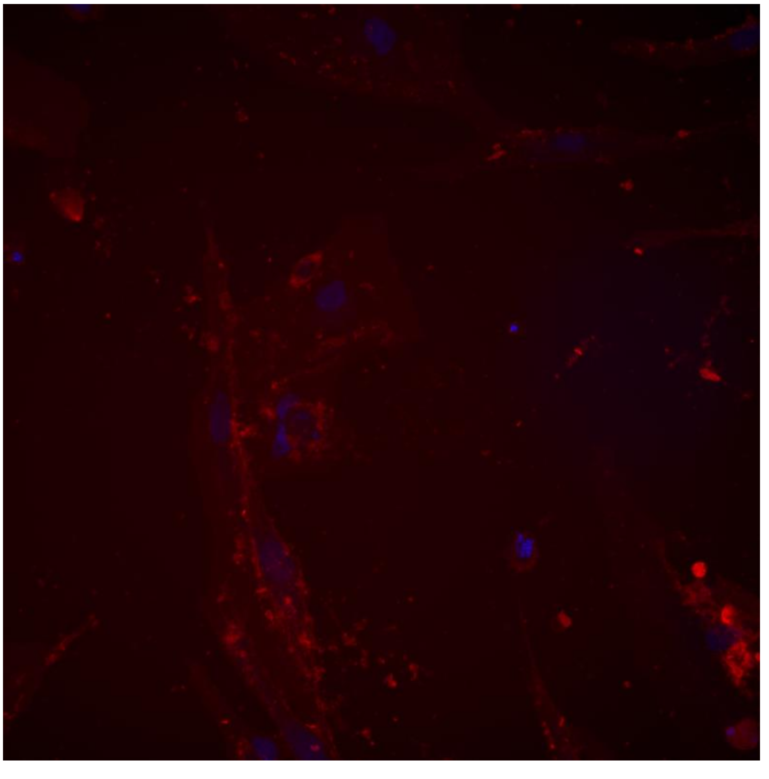

Supplement: Document S1. Figures S1–S3, Tables S2–S7, and Data S1–S3 [file mmc1.pdf]
